# Influence of batch effect correction methods on drug induced differential gene expression profiles

**DOI:** 10.1186/s12859-019-3028-6

**Published:** 2019-08-22

**Authors:** Wei Zhou, Karel K. M. Koudijs, Stefan Böhringer

**Affiliations:** 1 Department of Biomedical Data Sciences, Leiden University Medical Center, Leiden, The Netherlands; 2 Department of Clinical Pharmacy & Toxicology, Leiden University Medical Center, Leiden, The Netherlands; 3 Department of Internal Medicine, Erasmus Medical Center, Rotterdam, The Netherlands

**Keywords:** Drug repositioning, Batch effect, Microarray

## Abstract

**Background:**

Batch effects were not accounted for in most of the studies of computational drug repositioning based on gene expression signatures. It is unknown how batch effect removal methods impact the results of signature-based drug repositioning. Herein, we conducted differential analyses on the Connectivity Map (CMAP) database using several batch effect correction methods to evaluate the influence of batch effect correction methods on computational drug repositioning using microarray data and compare several batch effect correction methods.

**Results:**

Differences in average signature size were observed with different methods applied. The gene signatures identified by the Latent Effect Adjustment after Primary Projection (LEAPP) method and the methods fitted with Linear Models for Microarray Data (*limma*) software demonstrated little agreement. The external validity of the gene signatures was evaluated by connectivity mapping between the CMAP database and the Library of Integrated Network-based Cellular Signatures (LINCS) database. The results of connectivity mapping indicate that the genes identified were not reliable for drugs with total sample size (drug + control samples) smaller than 40, irrespective of the batch effect correction method applied. With total sample size larger than 40, the methods correcting for batch effects produced significantly better results than the method with no batch effect correction. In a simulation study, the power was generally low for simulated data with sample size smaller than 40. We observed best performance when using the *limma* method correcting for two principal components.

**Conclusion:**

Batch effect correction methods strongly impact differential gene expression analysis when the sample size is large enough to contain sufficient information and thus the downstream drug repositioning. We recommend including two or three principal components as covariates in fitting models with *limma* when sample size is sufficient (larger than 40 drug and controls combined).

**Electronic supplementary material:**

The online version of this article (10.1186/s12859-019-3028-6) contains supplementary material, which is available to authorized users.

## Background

Drug repositioning is the process of finding new indications for existing drugs. If successful, it has advantages over de novo drug development in terms of potentially shorter development times, less costs and risks [1]. Facilitated by recent growth of high-throughput omics data, computational methods in drug repositioning have been developed, which provide researchers efficient routes to explore a large number of drugs and diseases simultaneously [2]. Many in silico drug repositioning approaches have been developed during the past decades, which can be broadly classified into target-based, expression-based, knowledge-based, chemical structure-based, pathway-based and mechanism of action-based [3]. Here, we focus on gene expression-based approaches which require gene expression signatures derived from the data itself and require little a priori knowledge on diseases or drugs. A gene expression signature is a set of genes that are significantly up- or down-regulated by certain biological process or pathological medical condition as compared to a control condition. A popular approach is to identify new indications for drugs based on their gene signature showing an opposite pattern of up−/down-regulation as compared to a disease signature [4]. This approach was piloted by the Connectivity Map (CMAP) project, in which a pattern matching algorithm was employed to rank the similarities between the query signature and the compound profiles called reference signatures [5]. Several studies have used this resource and applied a similarity based approach for drug repositioning [6–10]. For example, Sirota et al. integrated 164 drug compounds from CMAP and 100 diseases to predict novel therapeutic indications on signatures in drug-disease pairs, which have led to the discovery of cimetidine as a candidate treatment for lung adenocarcinoma [7]. As another example, Van Noort et al. utilized the gene expression profiles of more than 1000 drugs from CMAP and applied the inverse signature approach to identify anti-metastatic drugs for the treatment of colorectal cancer [11]. The follow-up database to CMAP is the Library of Integrated Network-based Cellular Signatures (LINCS) L1000 database [12], which has been recently used in signature-based drug repositioning [13].

Despite CMAP having been demonstrated to be valuable and successful, it still has some limitations. These include a limited number of cell-lines and the fact that batches were required to generate all the data. Both factors can lead to biased analyses and here we focus on batch effects. Batch effects are defined as technical variations that have been introduced by time varying external factors during handling of the samples or effects of sample handling itself. Such factors include various sources, such as personnel effects, environmental conditions, different experiment times, etc. [14], some of which can be minimized by careful experimental design, while some are impossible to be completely avoided in practice. Whether batch effects were properly adjusted for can potentially affect the validity of the generated gene signatures as well as the power of the analysis to find differentially expressed genes [15, 16]. Many batch effect correction methods have been developed and were reviewed by Lazar et al. [17]. COMBAT (combining batches of microarray data) applies Empirical Bayes estimation to adjust the mean and the variance by pooling information across multiple genes in order to perform gene-wise batch corrections for mean and variance [18], which is an example for methods focusing on mean adjustments. Guided PCA (gPCA) performs a model selection on batch indicators/covariates known to impact measurements which is interesting when study design is complex and many potential factors that can influence the measurement process have been recorded. RUV-2 (“Remove Unwanted Variation, 2-step”) makes use of negative control genes that are a priori known to be uncorrelated with the biological effects of interest to identify the factors associated with batch effects, and further adjusts for these factors [19]. While RUV-2 relies on the quality of the control genes selected, the Latent Effect Adjustment after Primary Projection (LEAPP) method was developed to statistically isolate the batch effects from biological effect of interest, which in essence means that control genes are automatically selected [20]. Surrogate variable analysis (SVA) explicitly tries to define a subspace orthogonal to the outcome variable on which a principal component analysis (PCA), or an analogous singular value decomposition (SVD), is computed. In spirit, therefore, SVA is almost identical to LEAPP which performs the same decomposition but uses a slightly different model. RUV-2, LEAPP and SVA rely on principal components (PCs), explicitly or implicitly, to describe batch effects and can potentially correct for complex and non-linear batch effects.

However, in many drug repositioning studies, gene expression profiles were directly used from either CMAP or LINCS without correcting for batch effects [3, 6, 7, 10, 13]. Otherwise, mean centering was used to correct for batch effects (Noort et al. [11]). Koudijs et al. corrected for batch effects by blocking on batch id [4]. The impact of batch effect correction methods on computational drug repositioning efforts using these data resources, and their final impact on downstream drug repositioning pipelines has not been analysed.

**Fig. 1:**
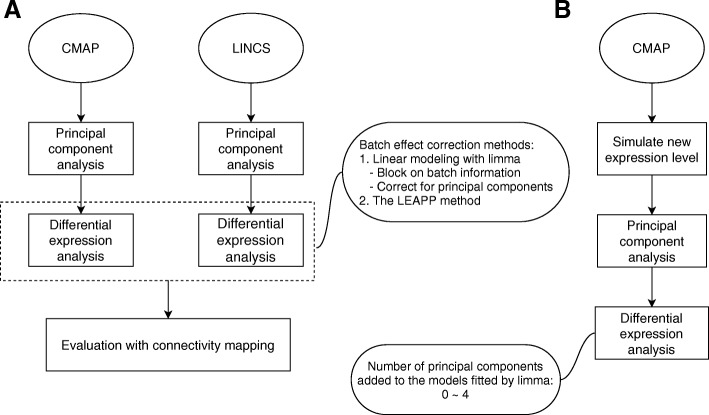
Overall workflow of the study. **a**, workflow of real data analysis. CMAP and LINCS datasets are analyzed by principal component analysis, followed by differential expression analysis with several batch effect correction methods, which were then evaluated by connectivity mapping (the procedure of connectivity mapping is illustrated by Additional file 2: Figure S2); **b**, workflow of simulation analysis. Expression data were simulated from CMAP dataset, and the optimal number of largest principal components being corrected for was assessed

In this study, we aim to investigate the influence of batch effect removal methods on computational drug repositioning focusing on microarray data, using the example of the CMAP dataset, since this is still the primary source of drug gene expression signatures. We conduct comparisons between several batch effect correction methods, including correcting for batch id and correcting for PCs in linear models fitted by *limma*, and the LEAPP method. We evaluate the quality of the gene signatures generated by these methods by gene set enrichment analyses on the shared drugs between the CMAP database and the LINCS database (Fig. 1a). We further perform a simulation study to examine the validity of the batch effect correction methods (Fig. 1b).

## Results

### Differential expression analysis

**Fig. 2:**
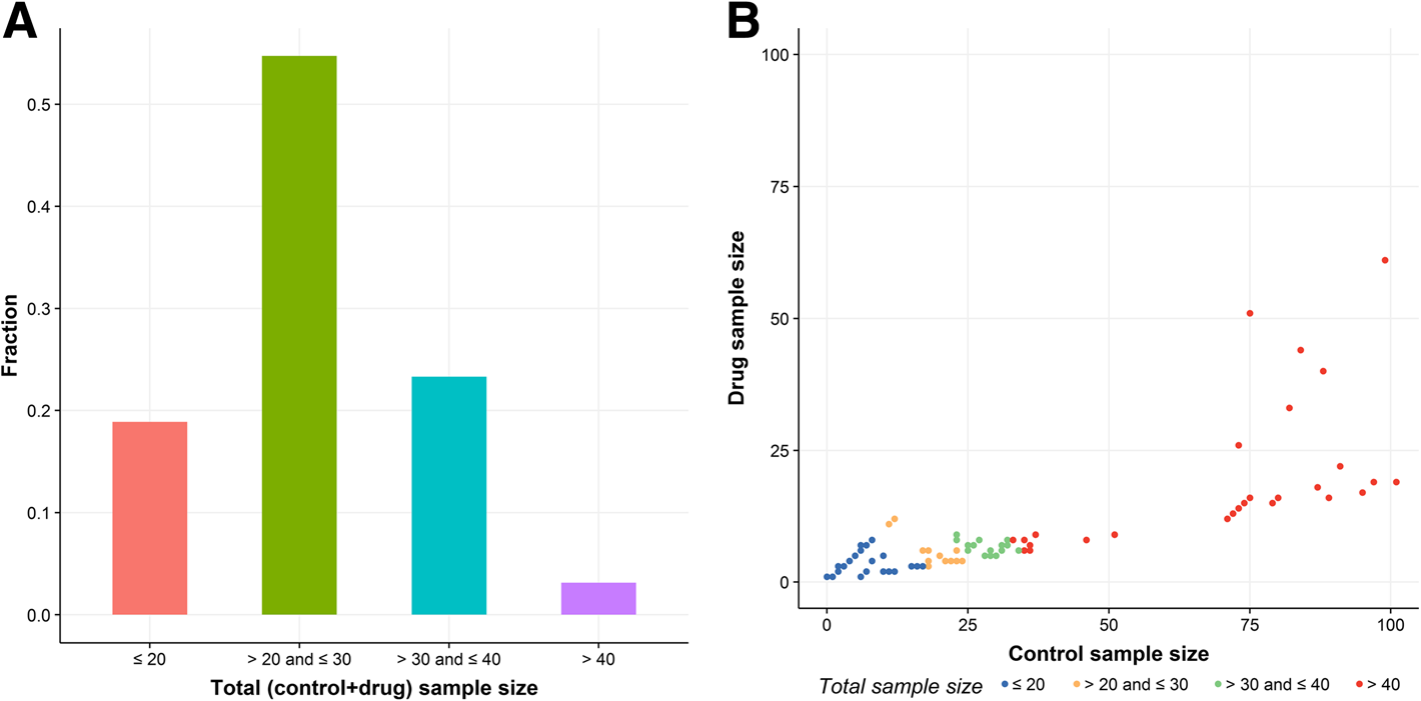
Summary plots of sample sizes in CMAP dataset. **a**, total (drug + control) sample size distribution in CMAP dataset. **b**, scatter plot of the relationship between control sample size and drug sample size for CMAP dataset. Note: the total number of drugs in CMAP dataset is 1309. In plot B, the drug trichostatin A (128 drug samples and 709 control samples) was not plotted because the particularly large sample size prevents a zoomed in view of other drugs

Figure 2 gives an overview of the distribution of sample sizes in CMAP dataset. Most of the drugs (55%) have total sample size between 20 to 30, while only a small fraction of drugs (3%) has total sample size more than 40. The scatter plot (Fig. 2b) shows that there are more control samples than drug samples for most of the drugs.

**Fig. 3:**
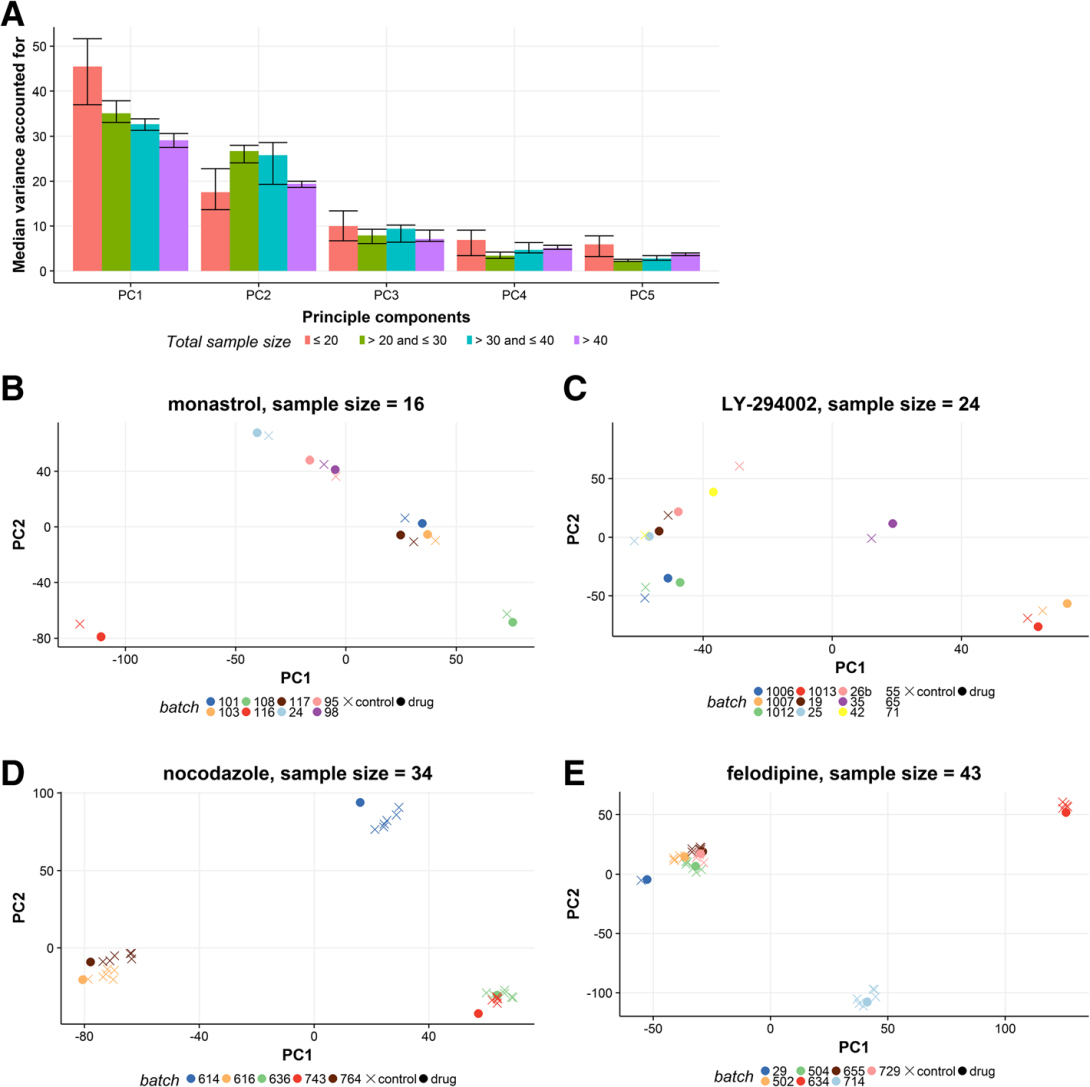
Results of principal component analysis on expression matrices for CMAP dataset. **a**, Median variance accounted for by the four largest principal components grouped by total sample size. **b**-**e**, Score plots of the first two principal components for four typical drugs; colors indicate batch (plate id) and shapes indicate drug or control status

Principal component analysis (PCA) was performed for every gene expression data matrix jointly for the treatment and control gene expression profiles corresponding to each drug. As is shown in Fig. 3a, the median variance explained by the first 2 PCs decreases with total sample size, from 62% (equal or below 20 samples) to 48% (above 40 samples). The samples clearly cluster by batch, but not by drug or control status (Fig. 3b-e). However, it should be noted that in CMAP batch and cell type are completely confounded for most drugs (Additional file 1: Figure S1).

Gene expression differences between drugs and vehicle controls were analyzed with linear models fitted by the *limma* package (version 3.32.5). The null model always contained the log-transformed concentrations. Subsequently, we tested if adding either the batch id or PCs improves the external validity of the genes identified as differentially expressed, as discussed below in the section on connectivity mapping. We included several sets of covariates to adjust for batch effects: i) null; ii) batch id (corresponding to the plate id); iii) one or more largest PCs (continuous variable). The linear associations between features and the drugs were also assessed by fitting models with the LEAPP method, for which no batch information was provided.

**Fig. 4:**
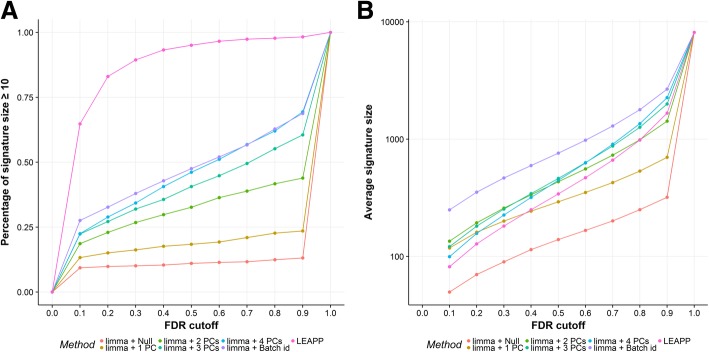
Results of differential expression analysis on CMAP dataset. **a**, percentage of drugs having signature size greater than or equal to 10 for each gene expression analysis method plotted against FDR cutoff. **b**, average signature size resulted from each gene expression analysis method plotted against FDR cutoff; y-axis was transformed to log-10 scale

After filtering out the genes with coefficients of variation outside the 20 and 80% quantiles, the number of genes included in the differential expression analysis decreased to 8131. Due to insufficient sample size, which did not allow some linear models to be fitted, some of the drugs do not have results produced in differential expression analysis, as illustrated in Table 1. The models fitted by LEAPP produced the largest percentages of results of drug signature size greater than or equal to 10 at any FDR < 100%, but the average signature size produced by the method was smaller than those generated by the methods respectively correcting for batch id, three or four PCs using *limma* (Fig. 4). Comparing among the methods using *limma*, correcting for batch id yielded largest percentages of results with drug signature size greater than or equal to 10 at any FDR ≤ 60%, followed by correcting for four PCs, and the percentages decreased with fewer PCs being included in the model (Fig. 4a). Similarly, the models fitted by *limma* with correction for batch id produced greatest average signature size and that with no correction yielded the smallest average signature size at any FDR < 100% (Fig. 4b). Table 2 summarizes the average number of shared differentially expressed genes generated by different methods for CMAP dataset at FDR ≤ 10%. In general, if two methods both show larger average signature size, they tend to share a higher percentage of shared genes as compared to other pairs of methods. Notably, we observed less agreement between the LEAPP method and the *limma* methods than the agreement between the methods that fit models using *limma* and use different sets of covariates. The LEAPP method resulted in many estimates that were exactly zero, even for genes that were considered statistically significant by LEAPP (FDR ≤ 10%), indicating numeric convergence problems, which prevented meaningful gene set enrichment analysis. Therefore, these results were not further analyzed.

**Table 1:** Number of drugs in CMAP dataset which yielded gene differential expression results by each method

Method	Number of results
limma+ Null	1288 (98.4%)
limma+1PC	1288 (98.4%)
limma+2PCs	1271 (97.1%)
limma+3PCs	1270 (97.0%)
limma+4PCs	1236 (94.4%)
limma + Batch id	1288 (98.4%)
LEAPP	1254 (95.8%)

Note: percentage out of 1309 drugs in parentheses

**Table 2:** Average number of shared differentially expressed genes found by different methods for the CMAP dataset (FDR ≤ 10%)

Method	limma+Null	limma+1PC	limma+2PCs	limma+3PCs	limma+4PCs	limma+Batch id	LEAPP
Limma+Null	(ASS = 50)	44 (37.3%)	27 (20.0%)	18 (14.9%)	14 (14.1%)	49 (19.6%)	9 (11.0%)
limma+1PC	44 (88.0%)	(ASS = 118)	61 (45.2%)	39 (32.2%)	28 (18.2%)	106 (42.4%)	15 (18.3%)
limma+2PCs	27 (54.0%)	61 (51.7%)	(ASS = 135)	81 (66.9%)	55 (55.6%)	120 (48.0%)	17 (20.7%)
limma+3PCs	18 (36.0%)	39 (33.1%)	81 (60.0%)	(ASS = 121)	70 (70.7%)	100 (40.0%)	17 (20.7%)
limma+4PCs	14 (28.0%)	28 (23.7%)	55 (40.7%)	70 (57.9%)	(ASS = 99)	75 (30.0%)	15 (18.3%)
limma+Batch id	49 (98.0%)	106 (89.8%)	120 (88.9%)	100 (82.6%)	75 (75.8%)	(ASS = 250)	25 (30.5%)
LEAPP	9 (18.0%)	15 (12.7%)	17 (12.6%)	17 (14.0%)	15 (15.2%)	25 (10.0%)	(ASS = 82)

Abbreviations: ASS = Average signature size (removed missing values)

Note: The table contains the number of differentially expressed genes that are shared between each pair of methods on the CMAP dataset. The numbers on the diagonal indicate the average number of differentially expressed genes found by the respective methods. For the LEAPP method, the significant genes with estimate = 0 were ignored. Percentages in parentheses are the proportions of the number of shared genes to average signature size produced by the method on the column header

### Connectivity mapping

**Fig. 5:**
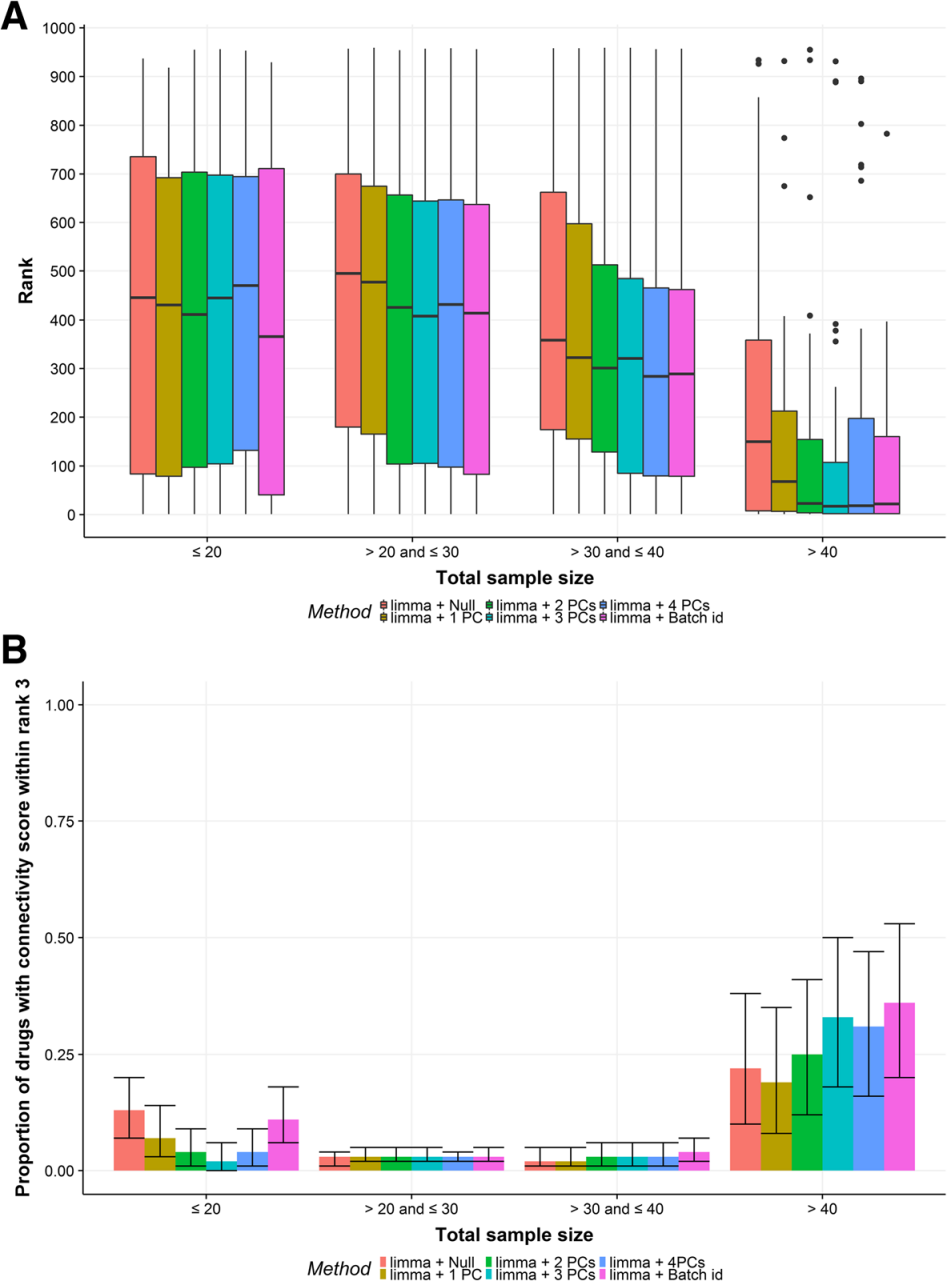
Results of connectivity score analysis with a fixed number of 15 genes with lowest FDR. **a**, Boxplot of the ranks of the same drug in connectivity mapping between CMAP and LINCS dataset. **b**, The proportion of drugs having the same drug ranked within top 3 in connectivity mapping between shared genes of CMAP and LINCS dataset. The x-axes are grouped by the total sample size in CMAP dataset. The colors indicate the differential gene expression analysis methods

To evaluate the batch effect correction methods on real data, as well as to mimic real practice drug repositioning utilizing gene expression-based approach, we used the CMAP drug signatures as input to identify the LINCS drug signatures using the relative connectivity score (with higher scores denoting higher similarities) calculated by Gene Set Enrichment Analysis using function ConnectivityScore implemented in PharmocoGx package [21]. For each comparison, the LINCS drug signatures were processed based on the drugs and the genes shared with the CMAP database using the same gene filtering criteria and the same batch effect correction method. If the method indeed improves the quality of the drug signatures, the relative rank of drug signatures of the same drug should increase after applying the method (Additional file 2: Figure S2). The LINCS dataset shares 962 drugs and 883 genes with CMAP dataset. After applying the same criteria of filtering, the overlapping number of genes used in differential expression analysis was 529. In the gene set enrichment analysis, when the gene set was limited to 15 genes with the lowest FDR values, the mean ranks of the drug signatures of the same drugs ranged between 250 to 500 in the groups of drugs with total sample size less than or equal to 40 for every method compared between CMAP dataset and LINCS dataset (Fig. 5a). The results improved dramatically in the group of drugs with total sample size greater than 40, in which the mean ranks ranged within 50 for *limma* methods correcting either for two, three or four PCs, or batch id (Fig. 5a). The methods correcting for two and three PCs and batch id were equivalent or significantly better than not correcting for batch effects or correcting for only one PC (*P* < 0.05). The superior performance of sample size > 40 is further demonstrated by the plot of the high proportion of drugs (19–36%) having the connectivity scores of the same drugs ranked within top 3 for every method stratified by group, compared to the low proportion of drugs within rank 3 (2–13%) observed in the group with sample size ≤40 (Fig. 4b). When the cut-off rank was relaxed from top 3 to top 10, similar results were obtained (Additional file 3: Figure S3).

We also performed the gene set enrichment analysis using sets of significant genes with FDR ≤ 10% and the results are plotted on Additional file 4: Figure S4A. In the group of drugs with small sample size, the method without batch effect correction resulted in higher proportions of drugs having the same drug ranked within top 3 in connectivity mapping between shared genes of CMAP and LINCS dataset.

An increasing trend was observed for the methods that correct for two to four PCs. When the cut-off rank was relaxed from top 3 to top 10, similar results were obtained (see Additional file 4: Figure S4B). Similar results were obtained for FDR cutoff at 5 and 20% (Additional file 5: Figure S5-Additional file 6: Figure S6). We emphasize that results shown in Additional file 4: Fig. S4, Additional file 5: Figure S5 and Additional file 6: Figure S6 only include the drugs that have at least 10 significant genes as indicated by the differential expression analysis, thus the drug lists varied among different methods and total sample size groups.

### Expression microarray data simulations

We simulated gene expression data from the original data of nine drugs with varying sample sizes, i.e. monastrol, LY-294002, colchicine, alprostadil, nocodazole, felodipine, vorinostat, fulvestrant and trichostatin A, of which the total sample size were 16, 24, 31, 33, 34, 43, 83, 128, and 837, respectively (Table 3). To address different situations, five simulation scenarios were applied and summarized in Table 4. These scenarios include different number of batches, allocation ratios and batch effect sizes (see Methods).

**Table 3:** Simulated drug profiles

Drug	Drug samples	Control samples	Total sample size	DEG in unsimulated data
monastrol	8	8	16	22
LY-294002	12	12	24	403
colchicine	6	25	31	21
alprostadil	7	26	33	18
nocodazole	5	29	34	1060
felodipine	7	36	43	72
vorinostat	12	71	83	5145
fulvestrant	40	88	128	1453
trichostatin A	128	709	837	6481

Note: drug, the drug of which the simulated data were generated from. DEG, differentially expressed genes, that is, the number of genes that were simulated to be differentially expressed due to the drug effects

**Table 4:** Simulation scenarios

Scenario name	Batch effect size parameter	FDR threshold	Batch allocation difference
No batch effect	0	0.1	0
Medium batch effect, balanced design	2	0.1	0
Large batch effect, balanced design	4	0.1	0
Medium batch effect, unbalanced design	2	0.1	0.3
Medium batch effect, balanced design, larger FDR threshold	2	0.2	0

Generally speaking, with more PCs added to the model, more significant genes were found regardless of the simulation setting applied (Additional file 7: Figure S7, Additional file 8: Figure S8, Additional file 9: Figure S9, Additional file 10: Figure S10 and Additional file 11: Figure S11A), although a few cases (vorinostat is especially exceptional across all the simulation settings) demonstrated first an increase, then a decreasing trend when considering different numbers of PCs with PC2 or PC3 as the turning point. The increased number of significant genes was at the cost of increased number of false positive results (Additional file 7: Figure S7, Additional file 8: Figure S8, Additional file 9: Figure S9, Additional file 10: Figure S10 and Additional file 11: Figure S11B). The proportions of false positive results were well controlled for, below or slightly higher than the pre-defined threshold for most of the simulated data when correcting for one or two PC(s). The only exception for this phenomenon was observed in the results of the data simulated from the drug colchicine, for which the highest proportions of false positive results were observed in the method without batch effect correction, and the proportions of false positive results were only well controlled in the setting of balanced batch design, medium batch effects and FDR at 10% when corrected for one or two PC(s) (Additional file 8: Figure S8). Notably, the number of simulated significant genes was small for this drug (Table 3). Moreover, for the data simulated from monastrol, which have a sample size smaller than 20, few significant results were obtained and proportions of false positives were extremely low, accordingly (Additional file 7: Figure S7, Additional file 8: Figure S8, Additional file 9: Figure S9, Additional file 10: Figure S10 and Additional file 11: Figure S11A-B).

Statistical power was generally lower than 20% for every method analyzed on the simulated data with total sample size smaller than 40, even without adding the additional simulated batch effects (Additional file 7: Figure S7, Additional file 8: Fig. S8, Additional file 9: Figure S9, Additional file 10: Figure S10 and Additional file 11: Figure S11C). For the data with total sample size larger than 40, with the increase of the total sample size, the statistical power increased, except for the data simulated from the real data of the drug fulvestrant.

Examining the simulation results of the data simulated from the real data of the drugs vorinostat and trichostatin A, we observed that: i) when the medium batch effects simulated from principal component loadings were added to the expression data, the power decreased by 10% for the method without batch effect correction, while the power of the methods correcting for two and three PCs only decreased by no more than 3% (Additional file 7: Figure S7 and Additional file 8: Figure S8C); ii) increasing of FDR value from 10 to 20% resulted in small increase in proportion of false positives (1–4%) in exchange for a higher increase in power (5–10%) (Additional file 8: Figure S8B-C and Additional file 11: Figure S11B-C); iii) compared to the results of the simulation with medium batch effect, when large batch effects were added to the data, the power of the method without batch effect correction and the method correcting for only one PC decreased substantially (> 15%), while the power of the method correcting for two and three PCs remained similar (< 5% difference) (Additional file 8: Figure S8C and Additional file 9: Figure S9C); iv) similar results were obtained when an unbalanced batch design was imposed (Additional file 8: Figure S8 and Additional file 10: Figure S10).

## Discussion

The present study investigated differential expression analyses with different batch effect correction methods on the publicly available datasets CMAP and LINCS. CMAP was used to obtain drug signatures, which are critical in downstream analyses of drug repositioning. The quality of the drug signatures generated by each method was further analyzed by connectivity mapping between the CMAP and the LINCS datasets on the subset of shared drugs and genes between the databases. Lastly, a simulation study was performed to compare models with different numbers of PCs included as covariates as well as the null models fitted by *limma*. To our knowledge, this study is the first to evaluate batch effects by conducting connectivity mapping between two datasets on shared drugs which can be seen as a gold-standard analysis, as the drugs should match up exactly if data is reliable.

We believe that our comparison covers at least conceptually a wide range of techniques employed in practice as many characteristics are shared among methods. Depending on whether batch information has to be explicitly specified or not, a method can be classified into being a supervised or unsupervised method. It is therefore critical that either batch information is correctly specified or a method can identify this information automatically. If the batch information is not well identified, the methods could under- or overcorrect depending on whether too little or too much information is used. The method correcting for batch id is the prototype of a supervised method that might undercorrect, as additional variations may be present within batches. PCA is an unsupervised batch effect correction method. As used in this paper, it might overcorrect as all genes were included in the estimation of PCs which includes those exhibiting true biological effects. Finally, LEAPP is an unsupervised method that might be optimal if the method achieves to separate genes represent batches from genes exhibiting biological effect. Arguably most methods fall into these broader categories and our results allow to judge whether conceptual trade-offs translate into results from data analyses, and simulations.

We showed that batch effect correction methods had a significant impact on the results of the gene expression analysis, and because the disease signature is directly compared to the results of the drug signature in gene-expression based drug repositioning [4], the downstream analyses of drug repositioning will likely be compromised in the case of uncorrected batch effects in the drug signature. This was demonstrated by i) the existence of significant batch effects as illustrated by PCAs; ii) that the generated gene signature sizes varied substantially between different batch effect correction methods; and iii) that in the group of drugs with total sample size larger than 40, we observed significant improvement in the relative ranks for batch effect correction methods compared to the null model in the analysis of connectivity mapping with a fixed number of 15 genes, that is, we were closer to the truth with batch effect correction compared to no batch effect correction. Note that we also conducted the analysis of connectivity mapping with sets of significant genes to mimic the analysis in practice (Additional file 4: Figure S4, Additional file 5: Figure S5 and Additional file 6: Figure S6), however, the plots of the results should not be taken as comparisons between the methods, since each method and sample size group had different list of drugs being analyzed by the gene set enrichment analysis depending on whether the drug produced a sufficiently large enough signature size (≥ 10). Therefore, the results depend highly on the average quality of the drug signatures which varied among the methods and prevents a fair comparison. For example, it is highly likely that the average quality of the drug signatures produced by the method without batch effect correction was higher only because the drug signatures with higher quality of genes (of evident signals) were found by the method, and thus the proportion of successful discoveries was higher for the method. This is supported by the fact that the method without batch effect correction found the fewest drugs with at least 10 significant genes among all the methods (Fig. 4).

In connectivity mapping, we showed that most of the CMAP drugs of total sample size smaller than 40 are not retrievable from LINCS (not among the connectivity score rank top 3). Therefore, the drug signatures generated by the differential expression methods investigated in this study were probably not reliable when the total sample size was smaller than 40, in the sense that the drug signatures are probably unable to perform well in downstream analysis of drug repositioning, no matter whether batch effects were corrected for or not. This was also supported by the simulation results, where we observed extremely low power in every simulated data with total sample size smaller than 40. Therefore, we conclude that more than 40 total samples are needed to generate reliable drug signatures from CMAP data.

The LEAPP method was not effective in our analysis—at least in the way we used it—although the method is theoretically advantageous and convenient (Sun et al. [20]). The differential expression results generated by the LEAPP method show little agreement with the *limma* methods, but we were unable to validate the quality of the drug signatures generated by the method or determine if the method was better in analyzing the CMAP dataset than the *limma* methods. The LEAPP method generated many estimates being exactly 0 in both the CMAP and the LINCS datasets, which prevented us from running the gene set enrichment analysis. Most likely, sample size in our application was too low for LEAPP to work reliably but we did not investigate this hypothesis in detail. We were unable to run simulations for LEAPP as it was too time consuming.

Among the *limma* methods, correcting for two and three PCs performed equally well as correcting for batch id when analyzing data with large sample size, as was indicated by the analysis of connectivity mapping. Nevertheless, the method adjusting for PCs has the potential to outperform the method adjusting for batch id for the following reasons: i) PC scores are continuous, which could detect relatively small technical differences within batches, such as, the temperature gradient on plates, and thus could have benefits over categorical variables like batch id; ii) PCs can be directly generated from the gene expression data so that the researcher does not need to rely on accurate batch labels; iii) PCs can be analyzed in a more refined fashion. For example, control genes could be introduced in the analysis, as is applied in the RUV-2 method [19]. Secondly, non-linear relations could be introduced to the model with PCs accompanied by model selection of non-linear terms.

In the simulation study, correcting for two PCs achieved relatively higher power and fewer false positives than correcting for other numbers of PCs in the simulated data of sufficiently large total sample size. In gene set enrichment analysis, though, correcting for three PCs performed relatively better. Based on the results, we recommend correcting for two or three PCs in data with sufficiently large sample size.

The simulation study also suggested less conservative FDR cutoff value should be considered. We speculate that the increase of the FDR threshold could increase power with small trade-off on the proportions of false positives, which might improve the results in the gene set enrichment analysis.

It is likely that PCs are unstable when the sample size is small, which may be one of the reasons that it did not perform well in data with small sample size in our analysis. PC correction can be adapted by applying weights in PCA by borrowing information from other data, such as, data from the same batch or by shrinking the covariance matrix towards the identity matrix [22]. We here only investigated raw PCs and modifications will be studied in future research.

In our study, we performed the analysis of connectivity mapping between two databases on the shared drugs. On the one hand, we implemented the practical procedure of computational drug repositioning. On the other hand, we provided a method to evaluate the quality of the drug signatures generated by differential expression methods, where we sought to find the same drugs back in the top of the lists ordered by the connectivity scores. Because the same drug is expected to affect the same cell line in different databases similarly, this could be considered a “gold standard”. However, there were also some limitations to this approach. Firstly, not all the cell types used in CMAP are available in LINCS. The analysis was done without matching the cell types between the two databases. Although ignoring the cell types may add noise to the analysis, the results are unbiased and robust. On the other hand, matching cell types would remove several samples from CMAP and thus take the analysis further away from real applications. Secondly, we imposed a fixed number of 15 genes in the signature for the analysis, which was rather small and may negatively affect results. The minimum number for gene set enrichment analysis was suggested to be 25 so as to avoid inflation of scorings [23]. We chose to standardize on 15 genes because most drugs could not identify at least 25 genes below the FDR cutoff. Thirdly, point estimates of fold changes were used to calculate the connectivity scores, which did not account for the uncertainties in the estimates. This could be addressed by for example weighting estimates according to *p*-values, or introducing another parameter determining the degree of weighting.

We observed that the power of the differential expression analyses on the data simulated from fulvestrant, which has a large total sample size (83), was extremely low. As can be seen in Additional file 12: Figure S12, the standard errors of the effect estimates of fulvestrant seem to be high, indicating a large noise component. The proportions of false positive results were not controlled at the pre-defined significance level in some cases despite the Benjamini Hochberg correction. No special patterns were observed in the histograms of the *P*-values for these cases (Additional file 13: Figure S13 and Additional file 14: Figure S14). Further research is needed to understand this phenomenon.

In the simulation study, we simulated both the case and control data under the null hypothesis by extracting the variance-covariance matrix from the real data of the vehicle controls, thereby capturing both biological and batch effects in the covariance matrix. Instead of interpreting such data as batch-effect free, we see it as a starting point for the simulations with a realistic covariance structure which is not necessarily identical to that of the actual drug. Moreover, the drug effects simulated were based on point estimates of differential expression analysis of real data which only reflect the truth up to uncertainty in estimates. The absence of further modifications of drug effects implies that some effects are overestimated and are exaggerated in the simulations. In the simulations, where the simulated drug effects were small, the power to detect the differences between simulated cases and simulated controls was expected to be small as well. Lastly, the batch effects were simulated from the first two PCs of the PCA, which is probably the reason that the method correcting for two PCs performed better than the other methods. On the other hand, the real data analysis supports the more general conclusion put forward in this discussion.

## Conclusions

Our study highlighted the importance of batch effect correction in computational drug repositioning, especially in generating gene expression signatures with the CMAP dataset, which has been used in at least 2800 studies. We recommend exercising caution in selecting proper batch effect correction methods. In applying the methods discussed in this study, sufficient sample size is essential to assure the validity of results. It is advisable to adjust for two or three PCs in the models fitted by *limma* when the total sample size is large enough (at least > 40 drug and controls combined), which applies to most of the drugs in LINCS (among the drugs shared with CMAP, 99.8% have total sample size larger than 40). However, for drugs of smaller total sample size, if analyzed with the methods discussed in this paper, the results should be interpreted with caution. Dealing with small sample sizes seems to require more method development.

Future work can include: i) applying weights and/or regularization in PCA on data with small sample size; ii) evaluating the optimal number of genes to be used in gene set enrichment analysis; iii) conducting simulations with various sizes of drug effects.

## Methods

### Data sources

CMAP database (build 2) was downloaded using the PharmacoGx package (version 1.6.1) [24]. Pre-processing of the database included Robust Multiarray Average (RMA) normalization, followed by correction for between platform differences using *combat* function in the SVA package (version 3.10.0) [25]. The CMAP dataset consists of 1309 distinct drugs. The number of genes in each gene expression profile is 11,833. In total, 7056 samples, including the bioactive perturbagens and their corresponding vehicle controls, were profiled. There are overall 302 batches, performed in five kinds of cell type.

The LINCS database in the level 3 format was obtained from the NCBI Gene Expression Omnibus (GEO) dataset (GSE92742), which was pre-processed by invariant set scaling and quantile normalization [12]. The number of genes provided at this level is 12,328 in total, out of which 11,350 were imputed from 978 landmark genes. However, we only included the 978 directly measured genes in our analysis. The samples profiled in the cell lines that had not been used in CMAP were excluded. Conversion from Entrez gene identifiers to Ensembl gene identifiers used by the CMAP database was performed using the bioMart package (version 2.32.1).

### Data cleaning

To avoid the effect of influential observations on the analyses, for every drug, samples were excluded if the concentration value used for the perturbation was more than 1.5 times the interquartile range above the third quartile or below the first quartile of the concentration values. The vehicle controls, i.e. samples containing only solvents for the active drug, from the same batches as the excluded drug samples were excluded as well. The total sample size of certain drug is therefore the sum of the number of drug samples and the number of the corresponding vehicle controls after exclusion.

To reduce the computational burden of the analyses while increasing the statistical power, we applied non-specific gene filtering by removing genes with coefficients of variation outside the 20 and 80% quantiles (coefficient of variation is the ratio of the standard deviation to the mean).

### Principal component analysis

PCA was used both as a descriptive tool to evaluate the existence of batch effects and as a correction method [19]. Scores of the PCs were extracted, which were subsequently added as covariates up to the first four components into the differential gene expression models. Plotting the scores is a way of visualizing batch effects. This analysis was performed using the built-in R function *prcomp*.

### Differential expression analysis

Concentrations of the vehicle controls were set to zero, while the concentrations of the drugs were rescaled to molar concentrations, and subsequently log_e_ plus one transformed (i.e. the mean of the log-transformed concentrations of vehicle controls was zero).

### Batch effect correction methods

#### Linear modeling with *limma*

*Limma* is an R/Bioconductor software package that fits linear model to each row that represents a gene in an gene expression matrix, as well as borrows information from the other genes analyzed, thus providing more reliable statistical results [26].

*The model without batch effect correction:*

$$ {y}_i={\beta}_0+{\beta}_1{X}_i+{\xi}_i, $$

where *y*_*i*_ is the expression value of sample i, *β*_0_ is the intercept, *β*_1_ is the drug effect, *X*_*i*_ is log_e_ plus one transformed molar concentrations of the drug, *ξ*_*i*_ is the residual for sample i.

We assessed the following batch effect correction methods that adjusted for covariates in linear models fitted by the *limma* package (version 3.32.5).

##### Blocking batch information in linear model

By including batch id (corresponding to the plate used to incubate the sample) while fitting linear model, this method adjusts the mean of the expression levels by the contrast of a batch with the reference batch.

$$ {y}_i={\beta}_0+{\beta}_1{X}_i+{\beta}_2{Z}_{1i}+\dots +{\beta}_{j+1}{Z}_{ji}+{\xi}_i, $$

where *y*_*i*_ is the expression value of sample i, *β*_0_ is the intercept, *β*_1_ is the drug effect, *X*_*i*_ is log_e_ plus one transformed molar concentrations of the drug, *β*_2_,..., *β*_*j* + 1_ are the coefficients of the dummy variables for batch IDs, *Z*_1*i*_, ..., *Z*_*ji*_ are the dummy variables for batch IDs (j indexes the batches) of sample i, *ξ*_*i*_ is the residual for sample i.

##### Correcting for principal components in linear model

The method adjusts for batch effect by including several PCs starting from the first as covariates while fitting the linear model. These PCs are believed to capture batch effects under the assumption that the variation caused by batch effects is much larger than the variation caused by drug effects. The method is similar to RUV-2 but without applying the PCA on negative control genes, as drug specific control genes have not been determined. The optimal number of PCs needed to capture the batch effect is part of the evaluation. In formula.

$$ {y}_i={\beta}_0+{\beta}_1{X}_i+{\beta}_2{C}_{1i}+\dots +{\beta}_{p+1}{C}_{pi}+{\xi}_i, $$

where *y*_*i*_ is the expression value of sample i, *β*_0_ is the intercept, *β*_1_ is the drug effect, *X*_*i*_ is log_e_ plus one transformed molar concentrations of the drug, *β*_2_,..., *β*_*p*_ are the coefficient of the principal components, *C*_1*i*_, ..., *C*_*pi*_ are the scores of the first 1 to p principal component(s) of sample i, *p* = 1, 2, 3 or 4, *ξ*_*i*_ is the residual for sample i.

Empirical Bayes procedures implemented in the *limma* package was employed to moderate estimated gene variances generated by *limma* models.

#### The latent effect adjustment after primary projection method

The LEAPP method attempts to automatically separate batch effects from the biological effects of interest by an estimation procedure. An attractive feature of the method is that it obviates the need of a list of control genes. The model estimates latent vectors corresponding to PCs so that residuals become uncorrelated, i.e. clustering in the data is removed. The number of latent variables is subject to variable selection and the method can be seen as PCA correction that searches control genes implicitly. The detailed description of the method can be found in the paper of Sun, et al. [20]. The analyses was conducted with LEAPP package (version 1.2). For the LEAPP function, we entered log_e_ plus one transformed concentrations as primary variables, assuming sparsity of the primary parameter. IPOD algorithm in Owen and She was applied to enforce sparsity [27]; hard thresholding was used in the algorithm to ensure robustness.

The resulting *P*-values were adjusted with Benjamini-Hochberg approach to control the false discovery rate (FDR). The significance level is defined at FDR 10% but other commonly used FDR levels (5, 20%) were also assessed.

### Connectivity mapping

The connectivity scores were calculated by Gene Set Enrichment Analyses (GSEA) with the function ConnectivityScore in PharmacoGx package (version 1.6.1) [28]. The Benjamini-Hochberg FDRs were recalculated for CMAP based on the genes shared with LINCS, after which the estimates of the 15 genes with lowest FDR values were extracted and compared to the corresponding set of genes in the LINCS database. For each drug in the CMAP dataset, we ranked the list of drugs in the LINCS dataset according to the order of the connectivity score from highest to lowest, and the rank of the corresponding same drug in LINCS dataset was extracted. Wilcoxon signed-rank test was used for comparing the resulting ranks between methods. Additionally, instead of using a fixed number of genes to calculate the connectivity scores, the same analysis was performed by only using the estimates of the differentially expressed genes defined by certain FDR threshold, so as to mimic the procedure of drug and disease connectivity mapping. The connectivity score was only calculated when the number of differentially expressed genes exceeded 9, which is the minimum required by the GSEA function. Different FDR cut-off values (FDR ≤ 5%, FDR ≤ 10% and FDR ≤ 20%) to determine significance were assessed.

### Expression microarray data simulations

Simulation studies were performed to compare i) the null model fitted by *limma*, and ii) the models fitted by *limma* with different number of PCs included as covariates. We based our simulated data on the correlation structure of real data corresponding to a representative selection of drugs and its vehicle controls. By simulating the same sample size as for the real data, simulations closely follow a realistic setting (significance level defined at FDR ≤ 10%). Simulated data under the null hypothesis were generated from real expression data of vehicle controls, with noise added by sampling from multivariate distribution with mean 0 and covariance matrix extracted block-wise from the data (1000 genes per block). Afterward drug effects extracted from the linear models fitted by *limma* on log-transformed drug concentration and adjusted for two PCs, were added to the simulated treatment group. Further, rescaled loadings of the first two PCs from the PCA on the real expression data of both the treatment and the control groups were used to simulate batch effects. The rescaling factors were 0, 2 and 4, representing no, medium and large batch effects respectively. The batch effects were simulated in four scenarios: 1) without loadings, 2) only the first principal component (PC1), 3) only the second principal component (PC2), and 4) PC1 and PC2. These four scenarios were always applied to the complete simulated case data, but the percentage applied to the control data thus modified depending on whether the batch effect was simulated as balanced or not. Thirty percent differences in batch allocation were imposed to simulate unbalanced designs. FDR cutoff values at 10 and 20% were evaluated. The simulation was conducted 10 times per drug and per setting.

## Additional files

Additional file 1: **Figure S1.** Score plots of the first two principal components for four typical drugs (A, B, C, D). Colors indicate batch (plate id) and shapes indicate cell type. (PDF 1116 kb)

Additional file 2: **Figure S2.** Using connectivity mapping to evaluate batch effect correction methods illustrated by ciclopirox. First, both CMAP and LINCS underwent the differencial expression analyses with the same batch effect correction methods, which resulted in drug signatures for all the drugs; second, the drug signature of ciclopirox in CMAP matched to all the drug signatures in LINCS, and the resulted connnectivity scores were ranked, where we expect that ciclopirox appears within the top three of the ranked list when the drug signature generated by the method is of high validity and good quality. (PDF 85 kb)

Additional file 3: **Figure S3.** Results of connectivity score analysis with a fixed number of 15 genes with the lowest FDR. The y axis is the proportion of drugs having the same drug ranked within top 10 in connectivity mapping between shared genes of CMAP and LINCS dataset. The error bars are the 95% confidence levels as estimated by binomial test. The x-axis is grouped by the total sample size in CMAP dataset. The colors indicate the differential gene expression analysis methods. (PDF 488 kb)

Additional file 4: **Figure S4.** Results of connectivity score analysis with all significant genes (FDR ≤ 10%). Only drugs with at least 10 significant genes yielded were included in the analysis. The y axis is the proportion of drugs having the same drug ranked within top 3 or 10 in connectivity mapping between shared genes of CMAP and LINCS dataset. The error bars are the 95% confidence levels estimated by binomial test. The x-axis is grouped by the differential gene expression analysis methods. The colors indicate the total sample size in CMAP dataset. (PDF 1461 kb)

Additional file 5: **Figure S5.** Results of connectivity score analysis with all significant genes (FDR ≤ 5%). Only drugs with at least 10 significant genes yielded were included in the analysis. The y axis is the proportion of drugs having the same drug ranked within top 3 or 10 in connectivity mapping between shared genes of CMAP and LINCS dataset. The error bars are the 95% confidence levels estimated by binomial test. The x-axis is grouped by the differential gene expression analysis methods. The colors indicate the total sample size in CMAP dataset. (PDF 1492 kb)

Additional file 6: **Figure S6.** Results of connectivity score analysis with all significant genes (FDR ≤ 20%). Only drugs with at least 10 significant genes yielded were included in the analysis. The y axis is the proportion of drugs having the same drug ranked within top 3 or 10 in connectivity mapping between shared genes of CMAP and LINCS dataset. The error bars are the 95% confidence levels estimated by binomial test. The x-axis is grouped by the differential gene expression analysis methods. The colors indicate the total sample size in CMAP dataset. (PDF 1433 kb)

Additional file 7: **Figure S7.** Results of the simulation study without batch effects and FDR < 10%. A, log10 transformed number of significant genes averaged over 10 simulations; B, Proportion of false positives among the significant genes averaged over 10 simulations; C. the power of the analysis averaged over 10 simulations. (PDF 95 kb)

Additional file 8: **Figure S8.** Results of simulation study with medium batch effects and FDR < 10%. A, log10 transformed number of significant genes averaged over 10 simulations; B, Proportion of false positives among the significant genes averaged over 10 simulations; C. the power of the analysis averaged over 10 simulations. (PDF 95 kb)

Additional file 9: **Figure S9.** Results of simulation study with large batch effects and FDR < 10%. A, log10 transformed number of significant genes averaged over 10 simulations; B, Proportion of false positives among the significant genes averaged over 10 simulations; C. the power of the analysis averaged over 10 simulations. (PDF 94 kb)

Additional file 10: **Figure S10.** Results of simulation study with medium batch effects, FDR < 10% and unequal allocation of cases and controls. Medium batch effect simulated with 0.3 differences between cases and controls. A, log10 transformed number of significant genes averaged over 10 simulations; B, Proportion of false positives among the significant genes averaged over 10 simulations; C. the power of the analysis averaged over 10 simulations. (PDF 94 kb)

Additional file 11: **Figure S11.** Results of simulation study with medium batch effects and FDR < 20%. A, log10 transformed number of significant genes averaged over 10 simulations; B, Proportion of false positives among the significant genes averaged over 10 simulations; C. the power of the analysis averaged over 10 simulations. (PDF 94 kb)

Additional file 12: **Figure S12.** Negative log 10 of *P*-values plotted against absolute estimates of extracted drug effects of felodipine, fulvestrant and vorinostat. (PDF 391 kb)

Additional file 13: **Figure S13.** Histograms of P-values resulted from differential expression analyses on one set of data simulated from colchicine with balanced batch design and median batch size (parameter settings see Table 4) at FDR ≤ 0.1. The differential expression analyses: A) limma + null model; B) limma + 1 PC; C) limma + 2 PCs; D) limma + 3 PCs; E) limma + 4 PCs. Abbreviations: PC, principal component; MPFP, mean proportion of false positive results. MPFP, mean proportion of false positives among the significant genes. (PDF 1648 kb)

Additional file 14: **Figure S14.** Histograms of P-values resulted from differential expression analyses on one set of data simulated from vorinostat with balanced batch design and median batch size (parameter settings see Table 4) at FDR ≤ 0.1. The differential expression analyses: A) limma + null model; B) limma + 1 PC; C) limma + 2 PCs; D) limma + 3 PCs; E) limma + 4 PCs. Abbreviations: PC, principal component; MPFP, mean proportion of false positive results. MPFP, mean proportion of false positives among the significant genes. (PDF 1242 kb)

## Data Availability

All data generated or analysed during this study are included in this published article. Software for simulation is available at https://gitlab.com/vivizhou/simulation_package.git.

## Abbreviations

CMAP: Connectivity Map
LEAPP: the Latent Effect Adjustment after Primary Projection
PCs: principal components
LINCS: Library of Integrated Network-based Cellular Signatures
PCA: principal component analysis
