# Supplementary figures and images for: Influence of batch effect correction methods on drug induced differential gene expression profiles
Source: BMC Bioinformatics. 2019 Aug 22;20:437. doi: 10.1186/s12859-019-3028-6 (PMC6706913; doi:10.1186/s12859-019-3028-6)

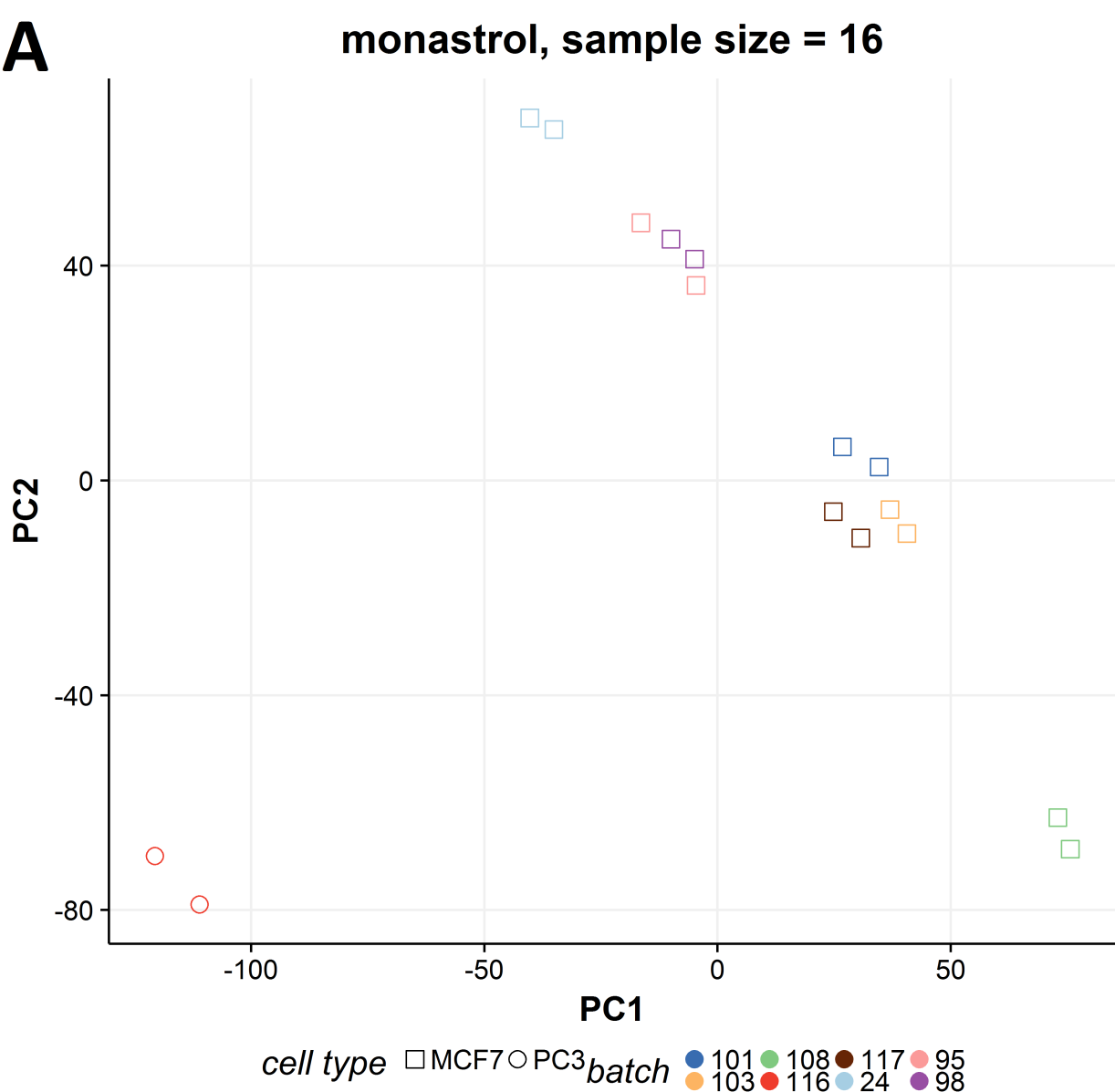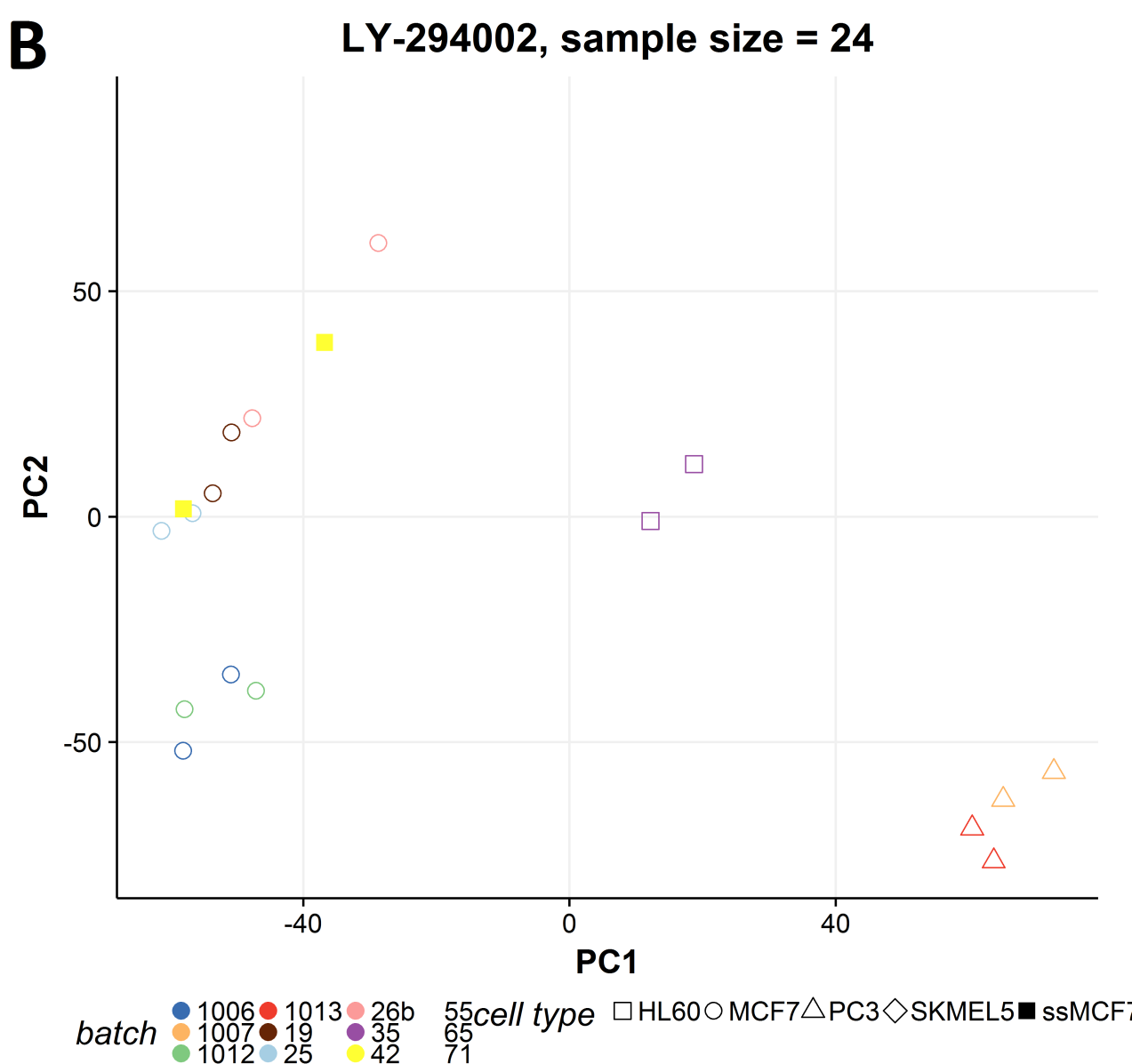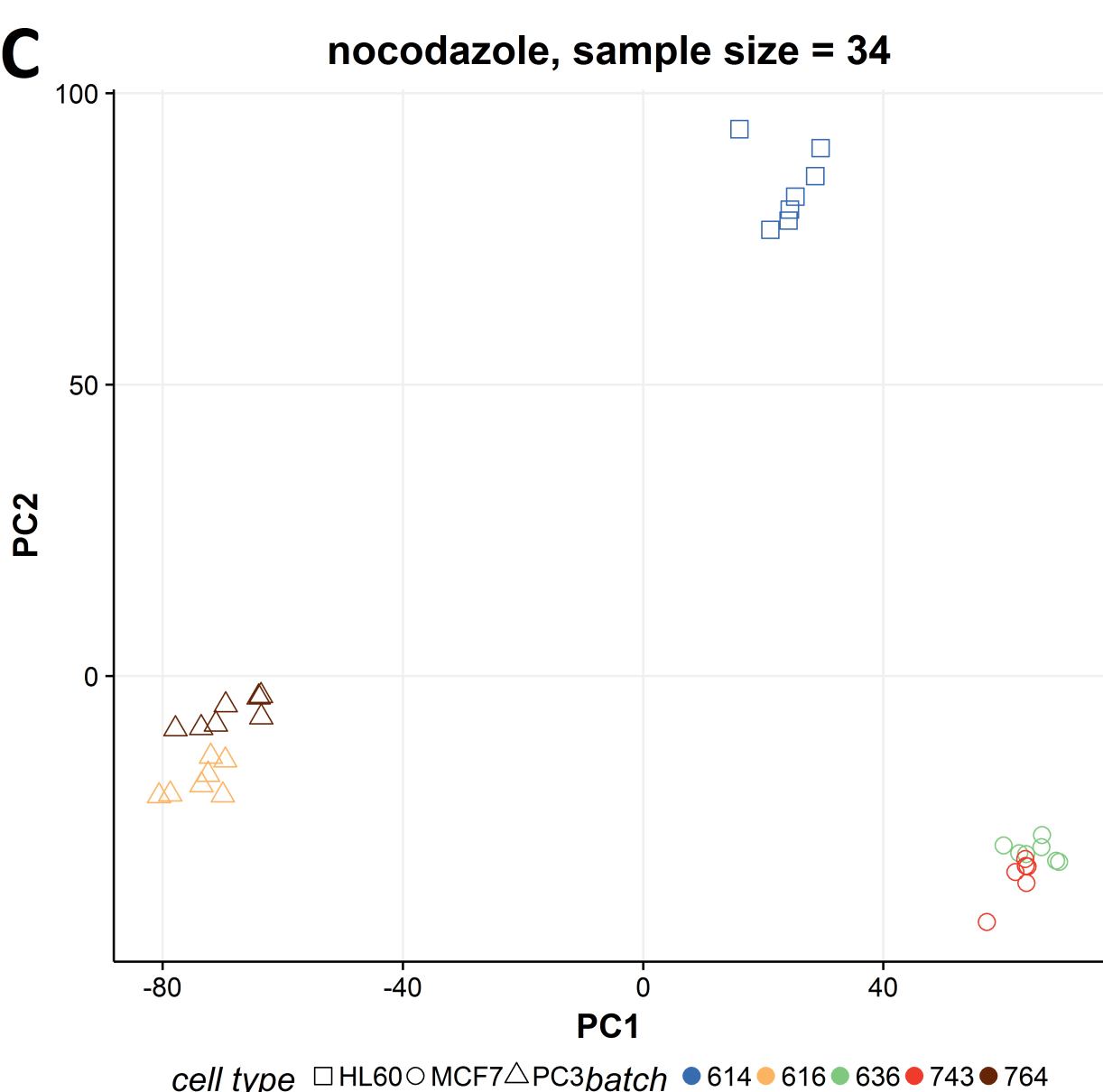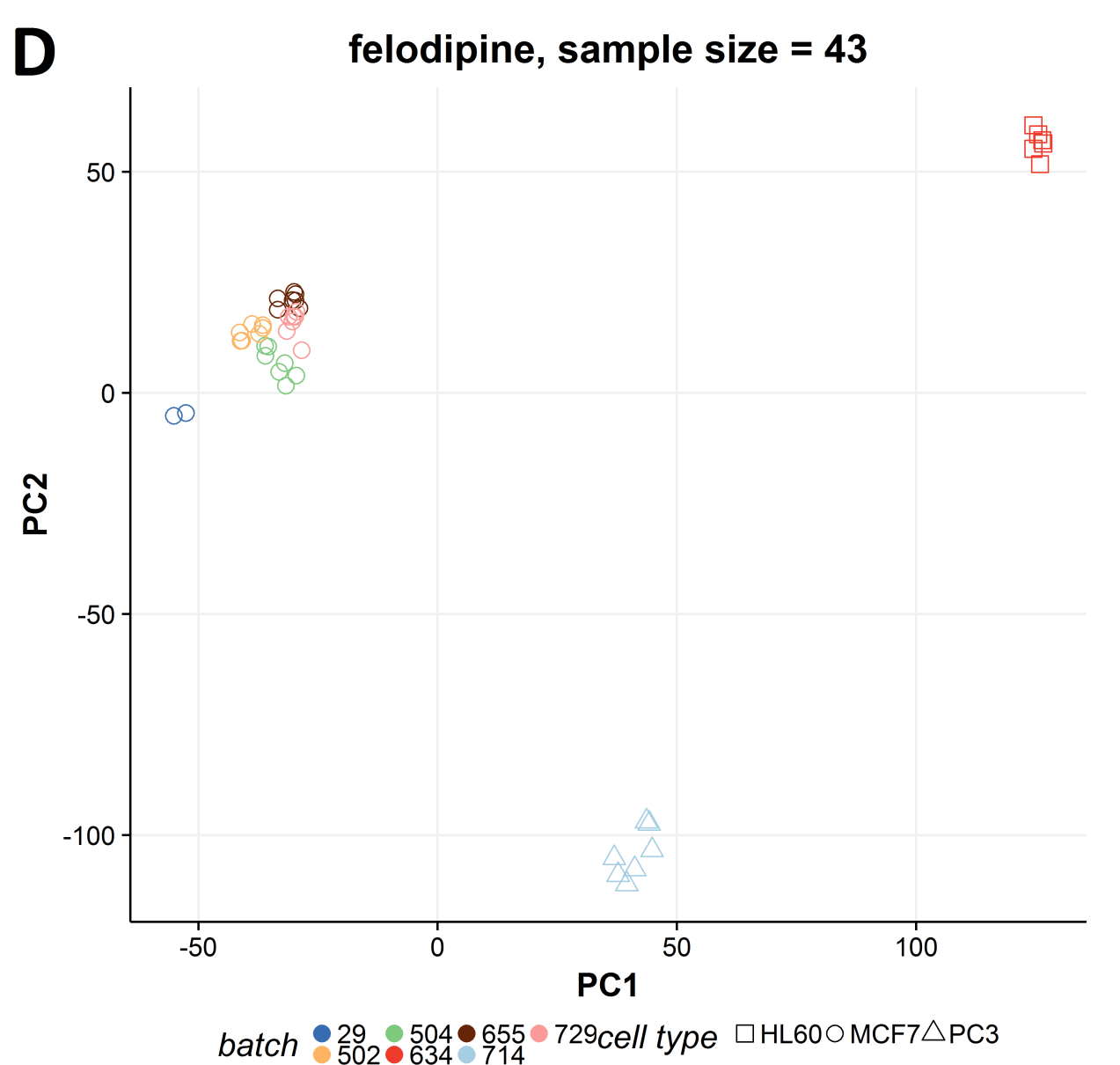

Supplement: Supplementary file 1 — Figure S1. Score plots of the first two principal components for four typical drugs (A, B, C, D). Colors indicate batch (plate id) and shapes indicate cell type. (PDF 1116 kb) [file 12859_2019_3028_MOESM1_ESM.pdf]

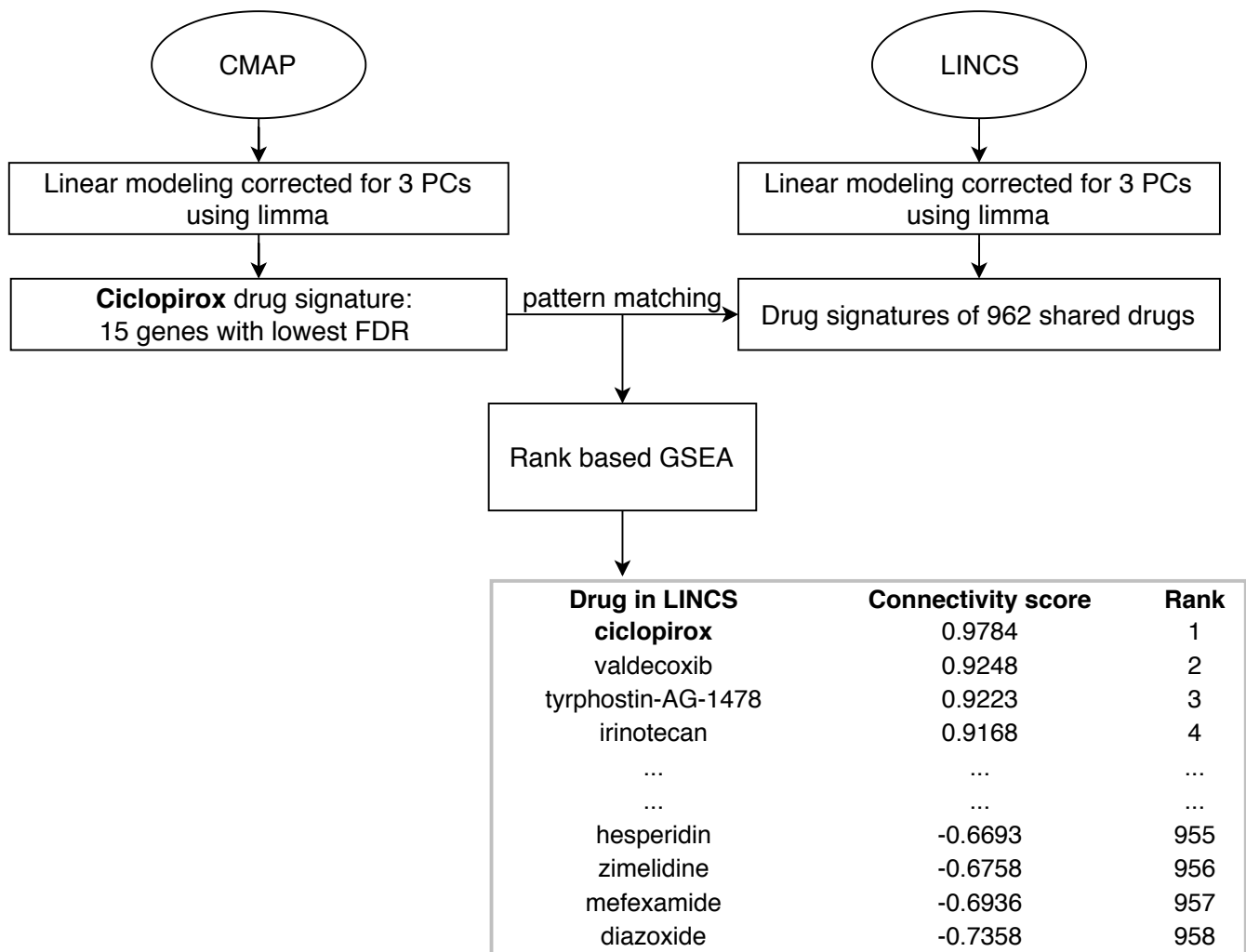

Supplement: Supplementary file 2 — Figure S2. Using connectivity mapping to evaluate batch effect correction methods illustrated by ciclopirox. First, both CMAP and LINCS underwent the differencial expression analyses with the same batch effect correction methods, which resulted in drug signatures for all the drugs; second, the drug signature of ciclopirox in CMAP matched to all the drug signatures in LINCS, and the resulted connnectivity scores were ranked, where we expect that ciclopirox appears within the top three of the ranked list when the drug signature generated by the method is of high validity and good quality. (PDF 85 kb) [file 12859_2019_3028_MOESM2_ESM.pdf]

Proportion of drugs with connectivity score within rank 10

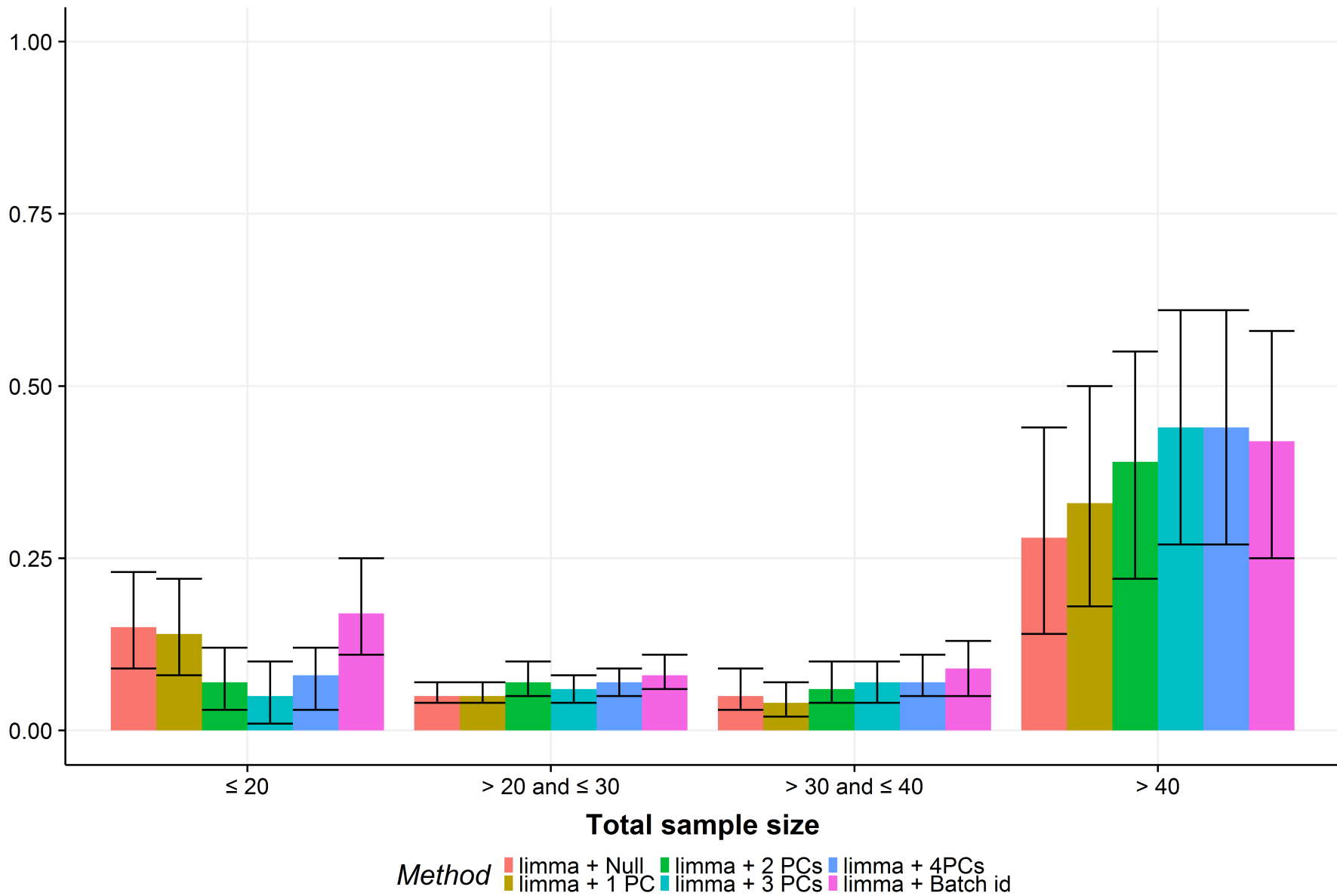

Supplement: Supplementary file 3 — Figure S3. Results of connectivity score analysis with a fixed number of 15 genes with the lowest FDR. The y axis is the proportion of drugs having the same drug ranked within top 10 in connectivity mapping between shared genes of CMAP and LINCS dataset. The error bars are the 95% confidence levels as estimated by binomial test. The x-axis is grouped by the total sample size in CMAP dataset. The colors indicate the differential gene expression analysis methods. (PDF 488 kb) [file 12859_2019_3028_MOESM3_ESM.pdf]

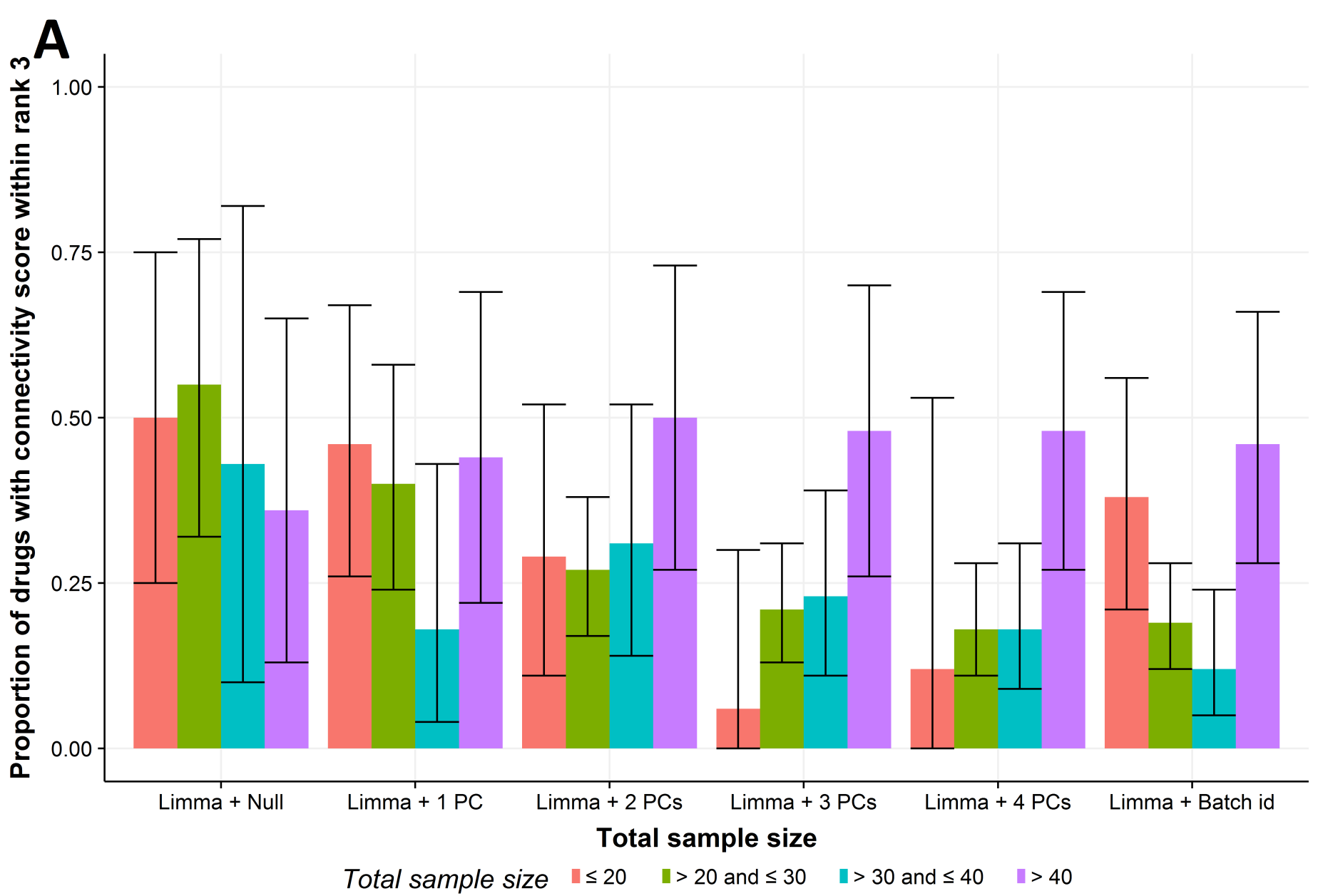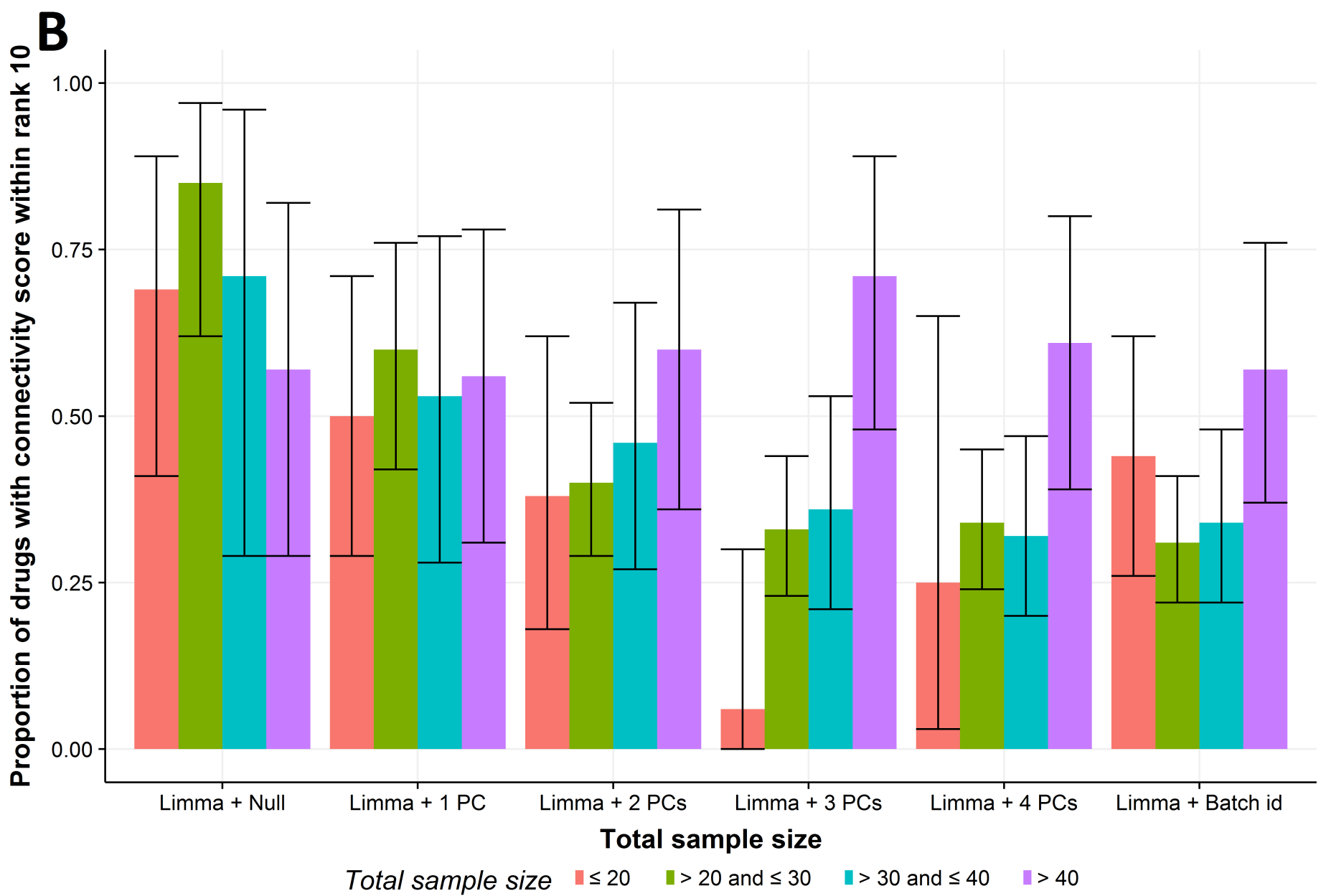

Supplement: Supplementary file 4 — Figure S4. Results of connectivity score analysis with all significant genes (FDR ≤ 10%). Only drugs with at least 10 significant genes yielded were included in the analysis. The y axis is the proportion of drugs having the same drug ranked within top 3 or 10 in connectivity mapping between shared genes of CMAP and LINCS dataset. The error bars are the 95% confidence levels estimated by binomial test. The x-axis is grouped by the differential gene expression analysis methods. The colors indicate the total sample size in CMAP dataset. (PDF 1461 kb) [file 12859_2019_3028_MOESM4_ESM.pdf]

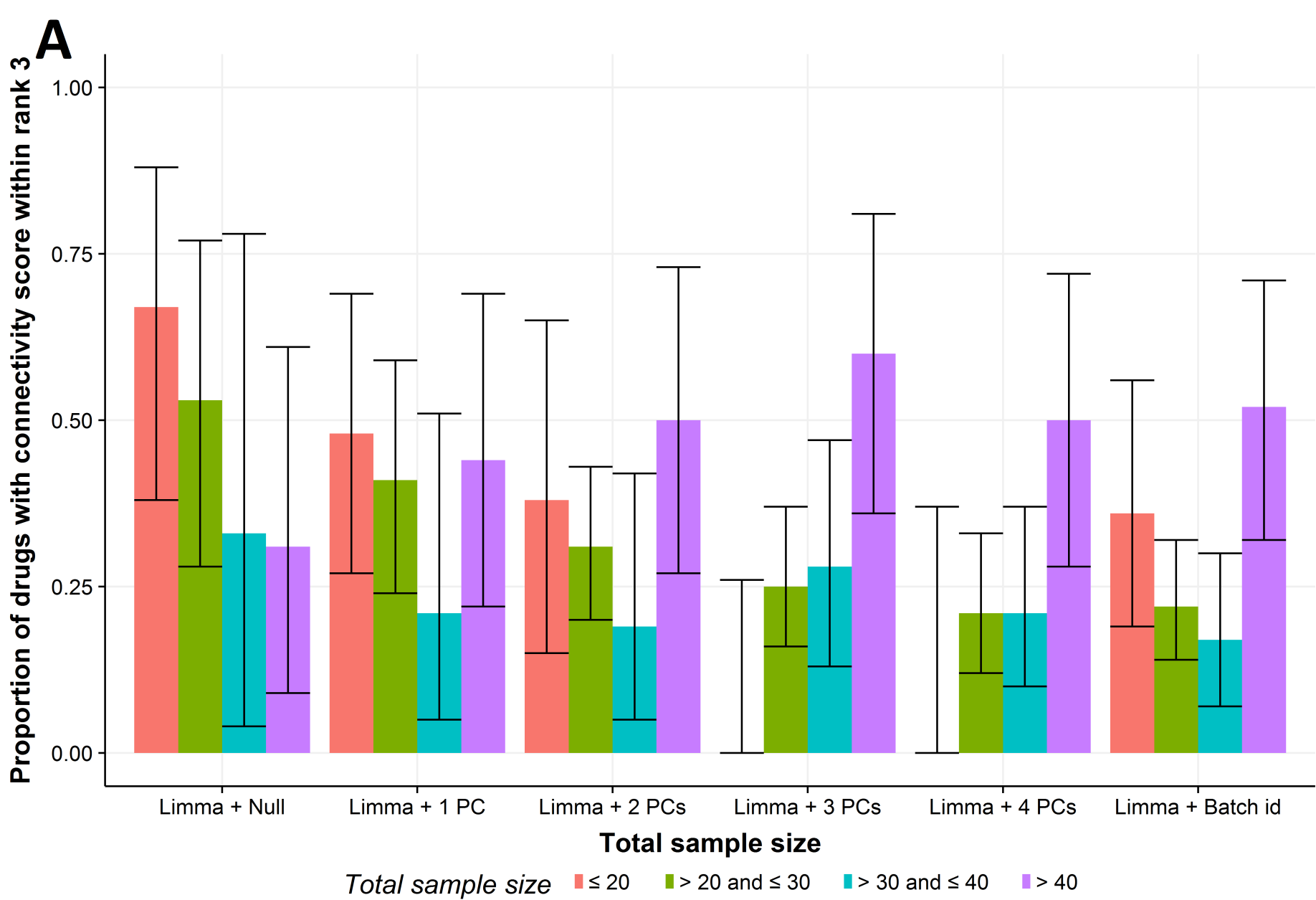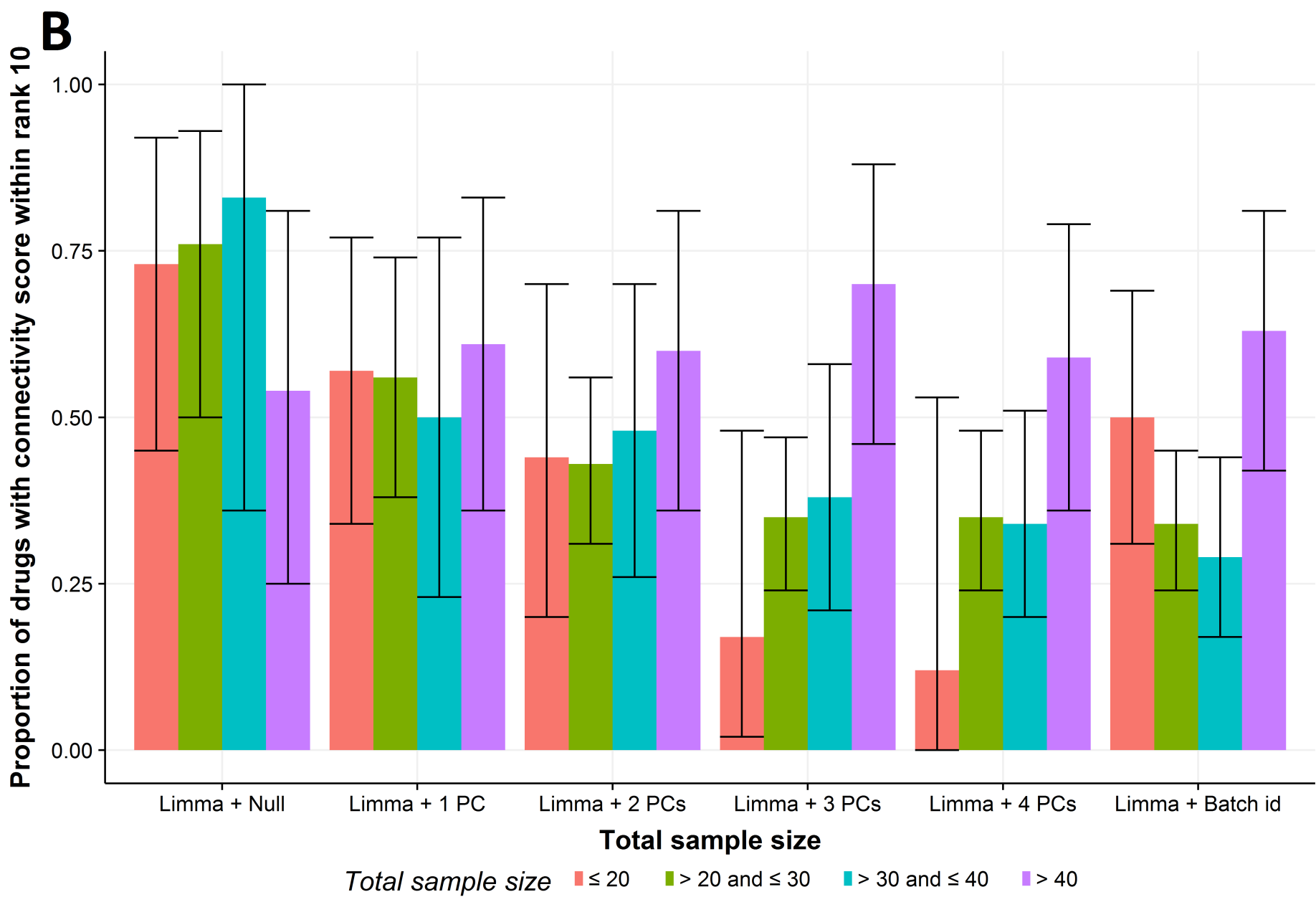

Supplement: Supplementary file 5 — Figure S5. Results of connectivity score analysis with all significant genes (FDR ≤ 5%). Only drugs with at least 10 significant genes yielded were included in the analysis. The y axis is the proportion of drugs having the same drug ranked within top 3 or 10 in connectivity mapping between shared genes of CMAP and LINCS dataset. The error bars are the 95% confidence levels estimated by binomial test. The x-axis is grouped by the differential gene expression analysis methods. The colors indicate the total sample size in CMAP dataset. (PDF 1492 kb) [file 12859_2019_3028_MOESM5_ESM.pdf]

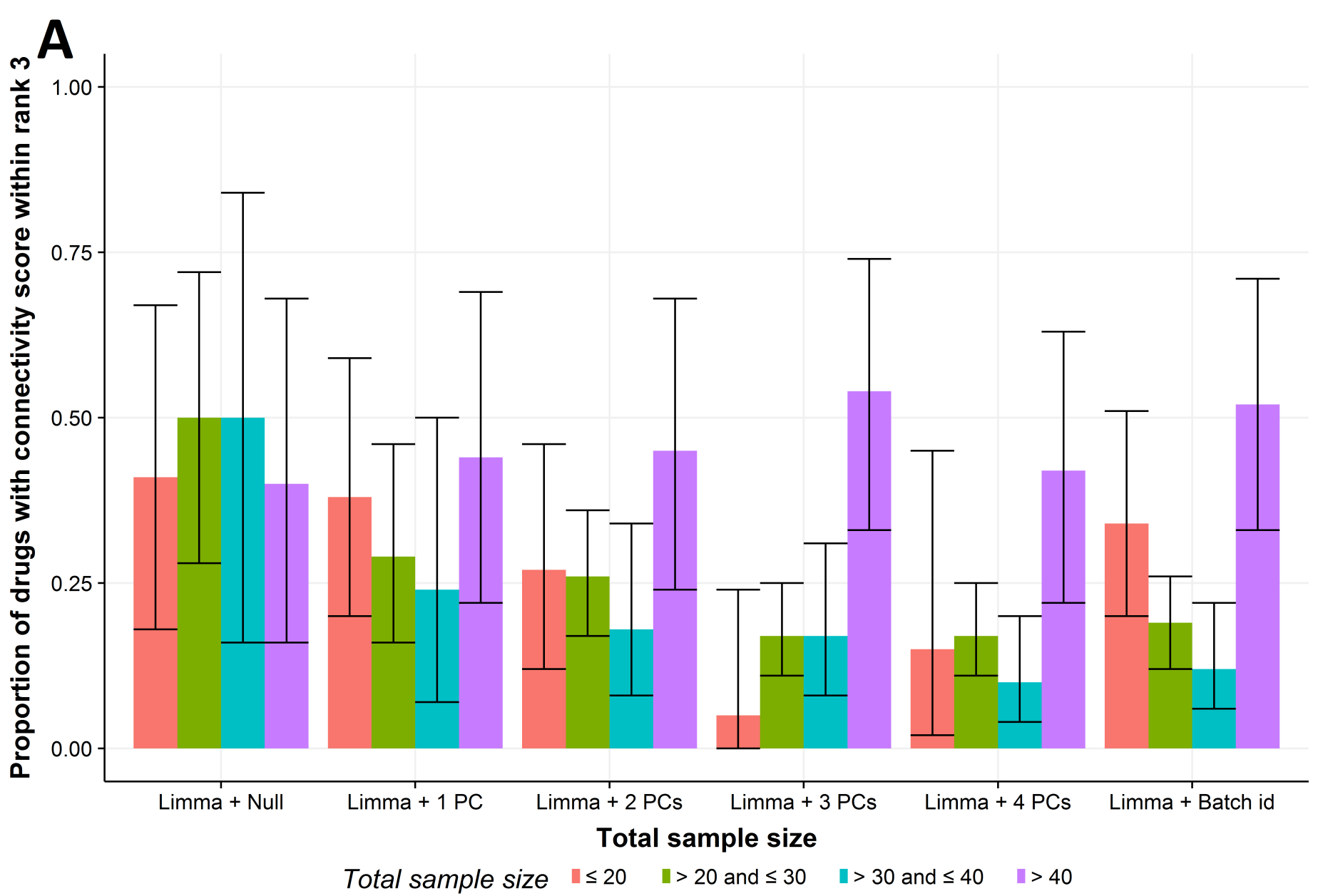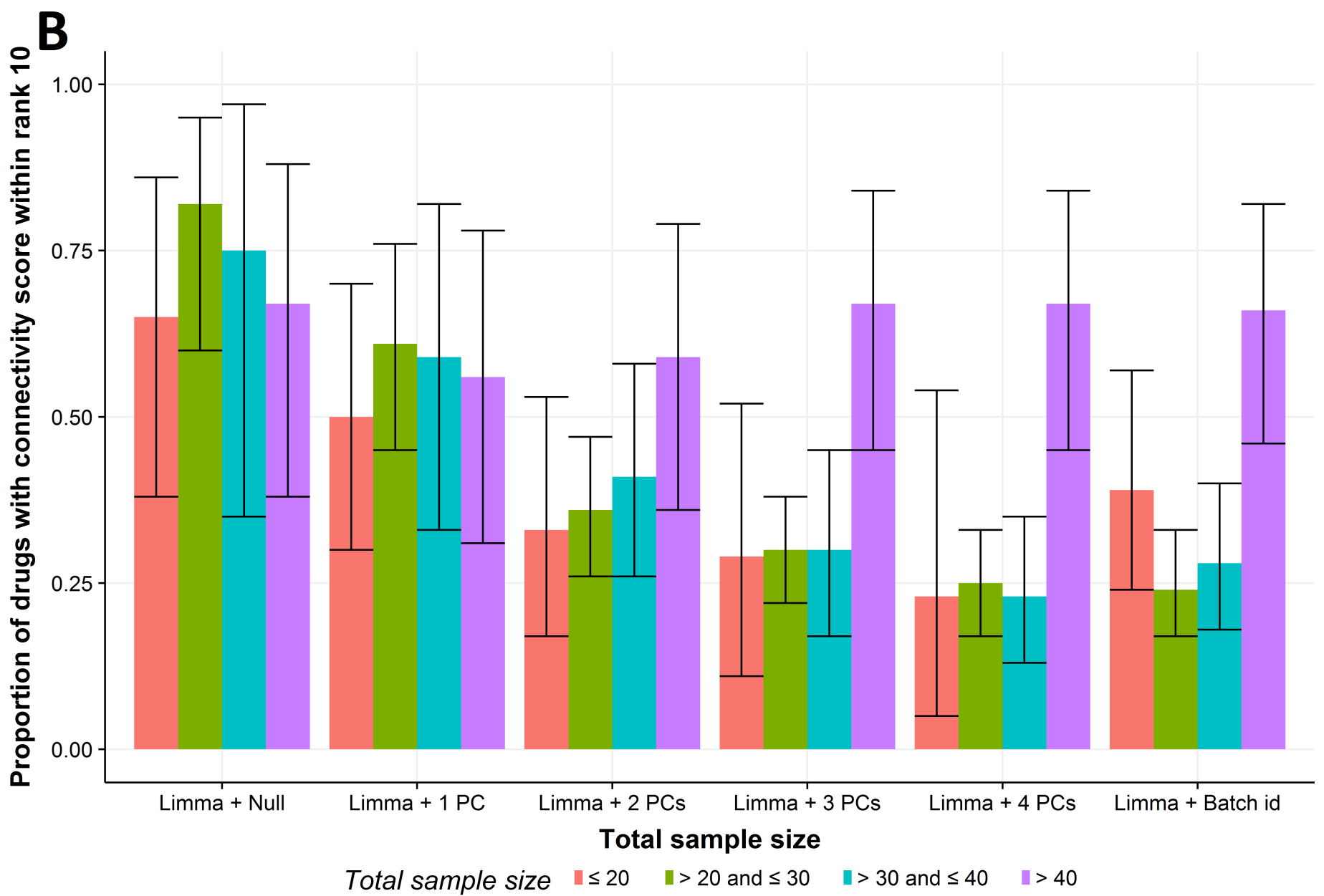

Supplement: Supplementary file 6 — Figure S6. Results of connectivity score analysis with all significant genes (FDR ≤ 20%). Only drugs with at least 10 significant genes yielded were included in the analysis. The y axis is the proportion of drugs having the same drug ranked within top 3 or 10 in connectivity mapping between shared genes of CMAP and LINCS dataset. The error bars are the 95% confidence levels estimated by binomial test. The x-axis is grouped by the differential gene expression analysis methods. The colors indicate the total sample size in CMAP dataset. (PDF 1433 kb) [file 12859_2019_3028_MOESM6_ESM.pdf]

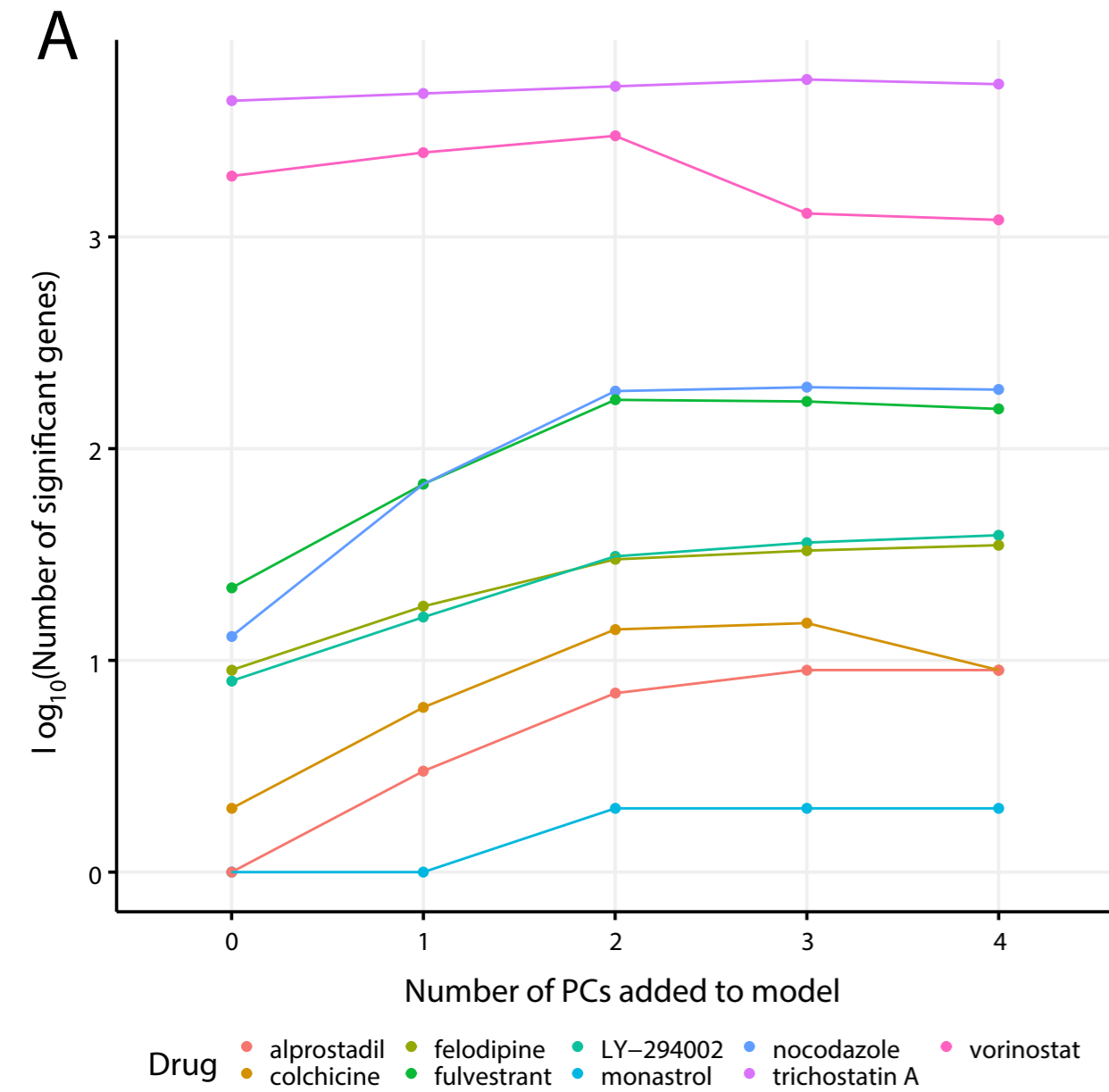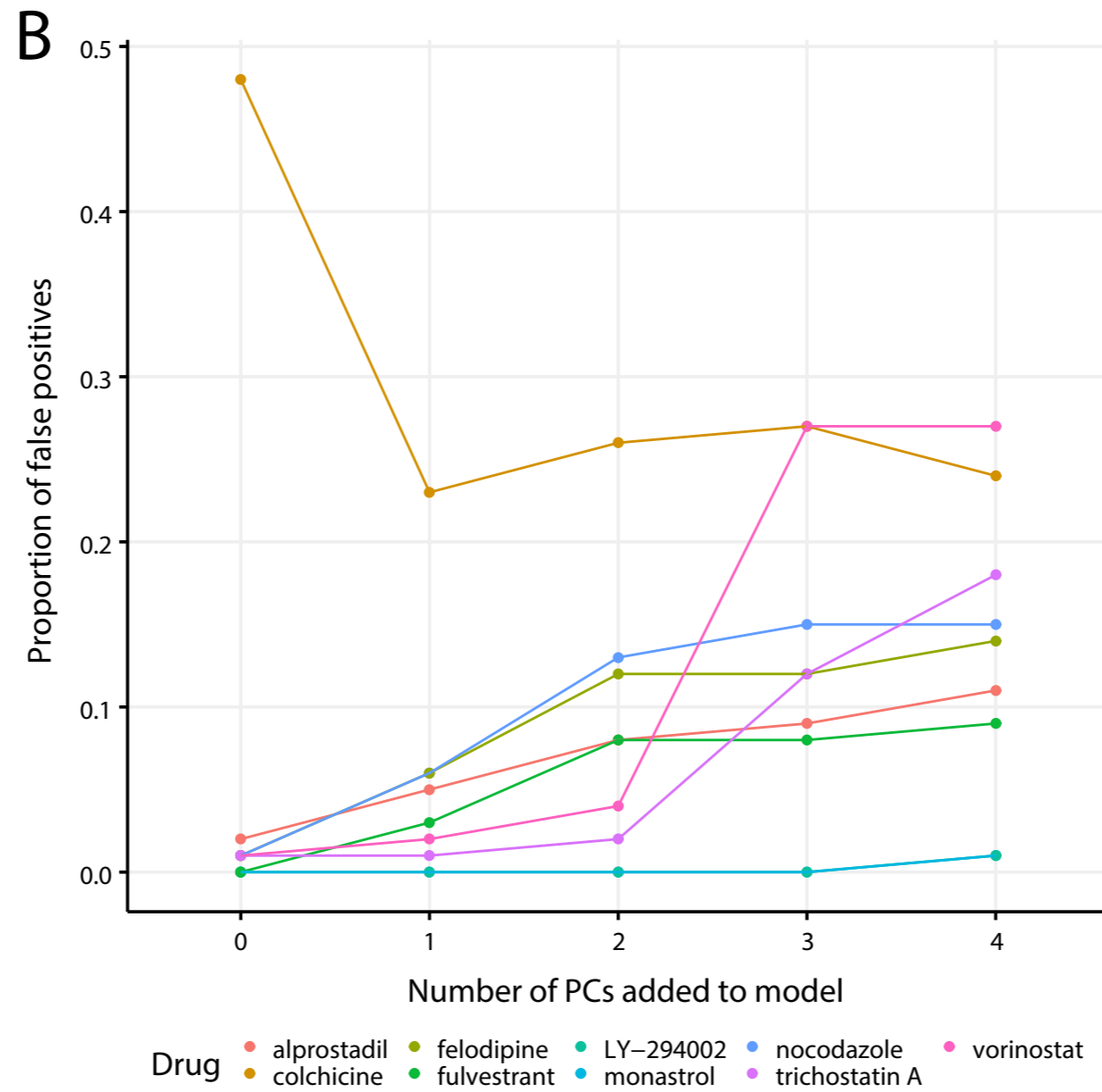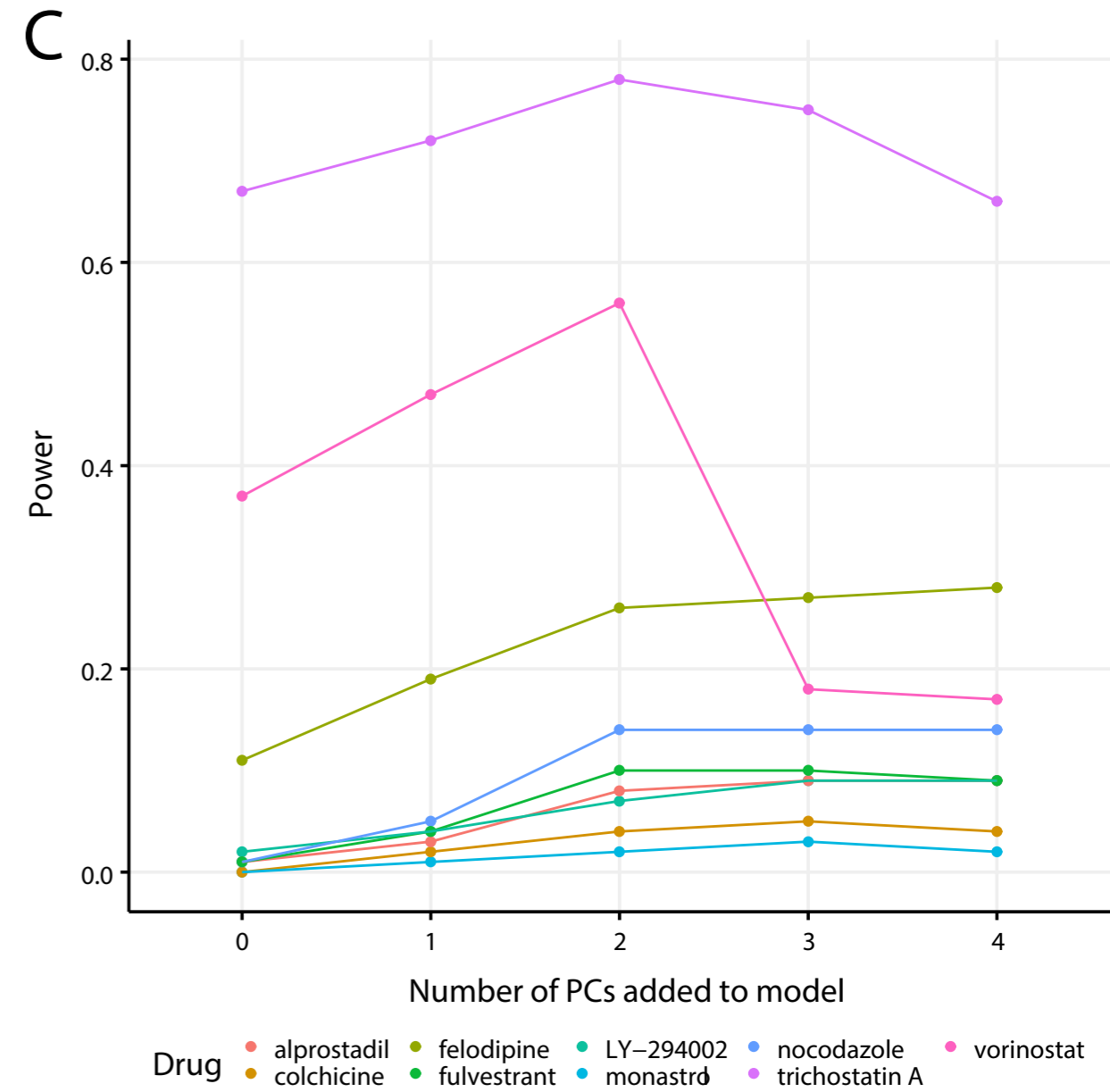

Supplement: Supplementary file 7 — Figure S7. Results of the simulation study without batch effects and FDR < 10%. A, log10 transformed number of significant genes averaged over 10 simulations; B, Proportion of false positives among the significant genes averaged over 10 simulations; C. the power of the analysis averaged over 10 simulations. (PDF 95 kb) [file 12859_2019_3028_MOESM7_ESM.pdf]

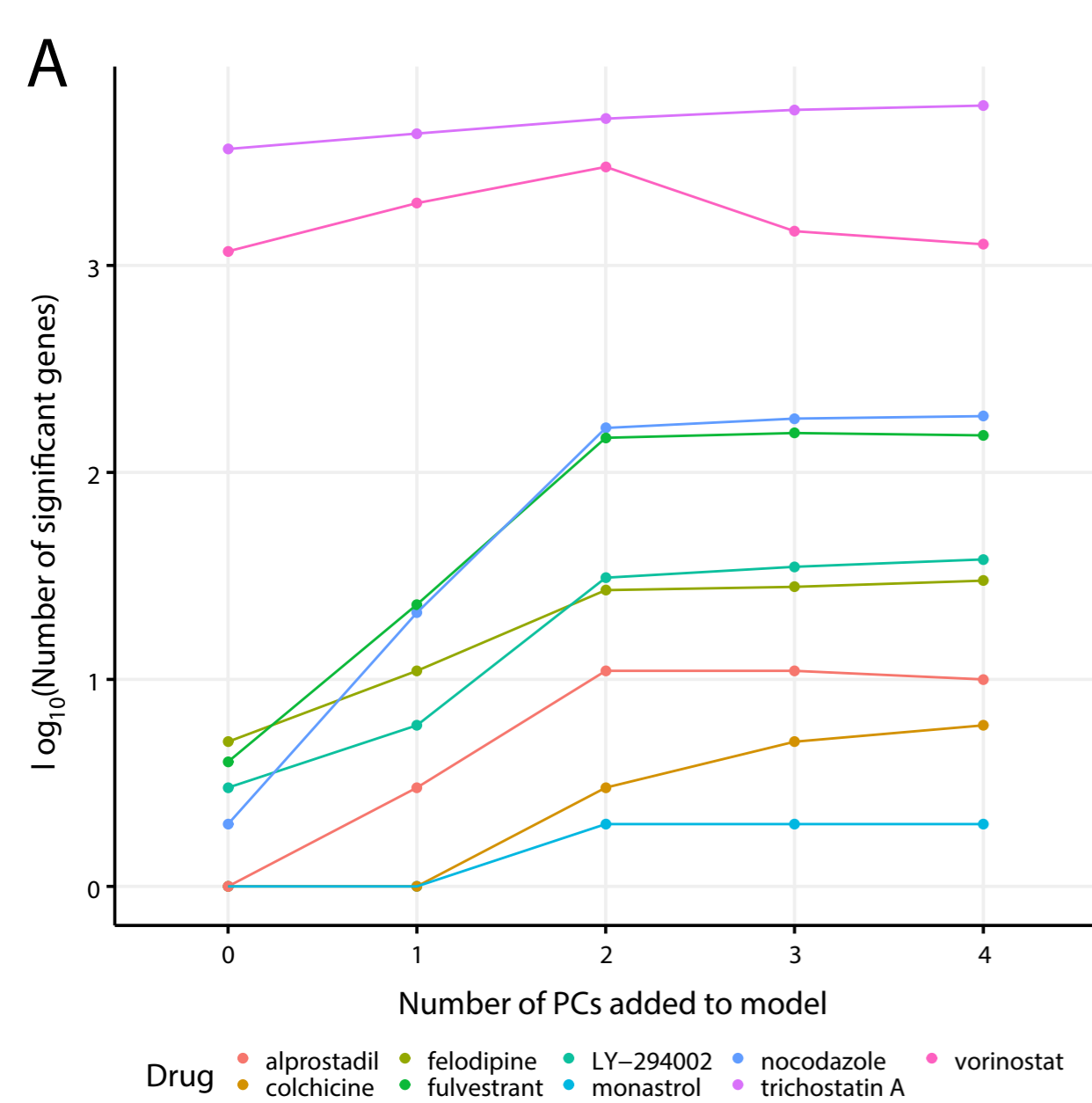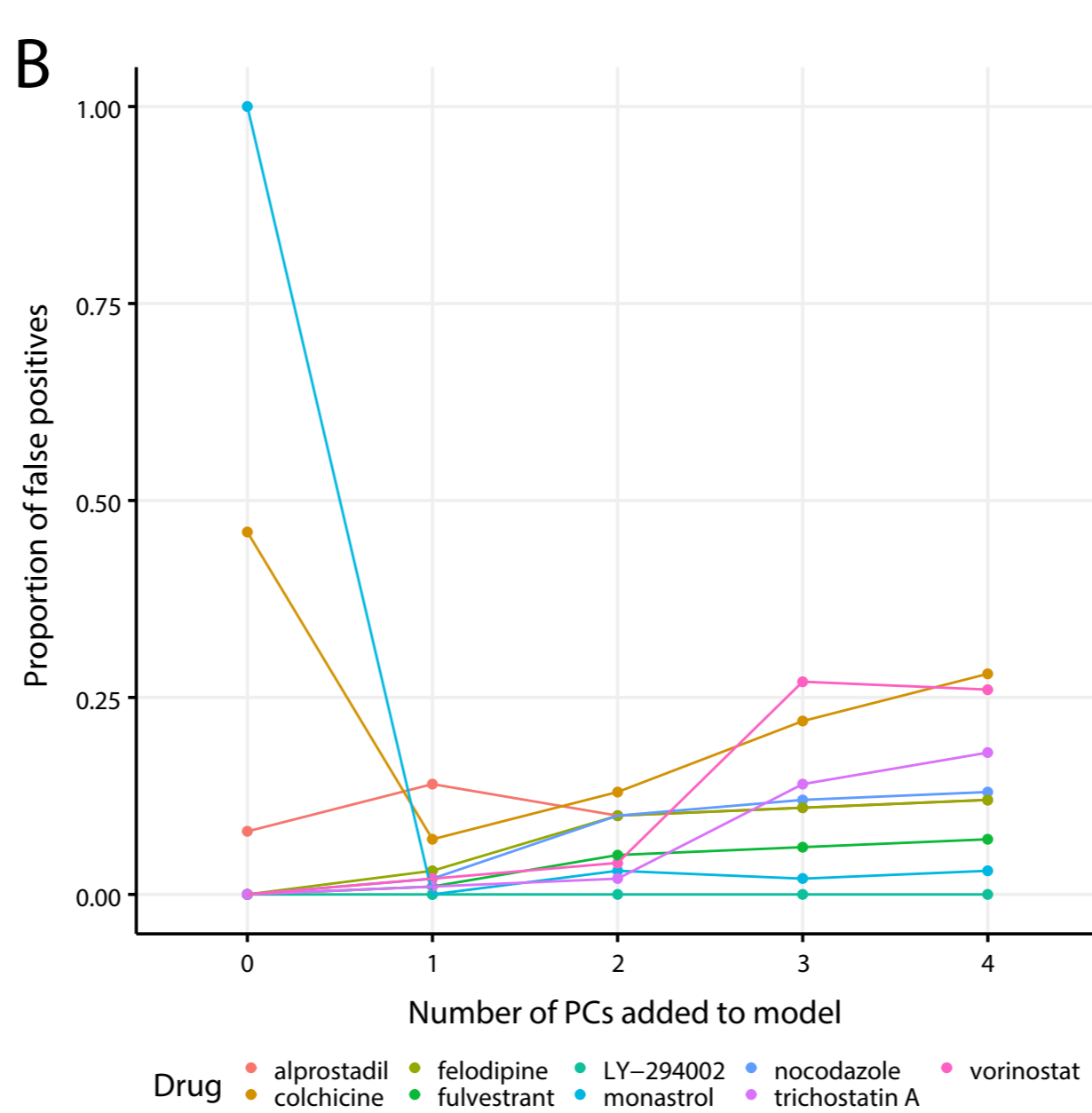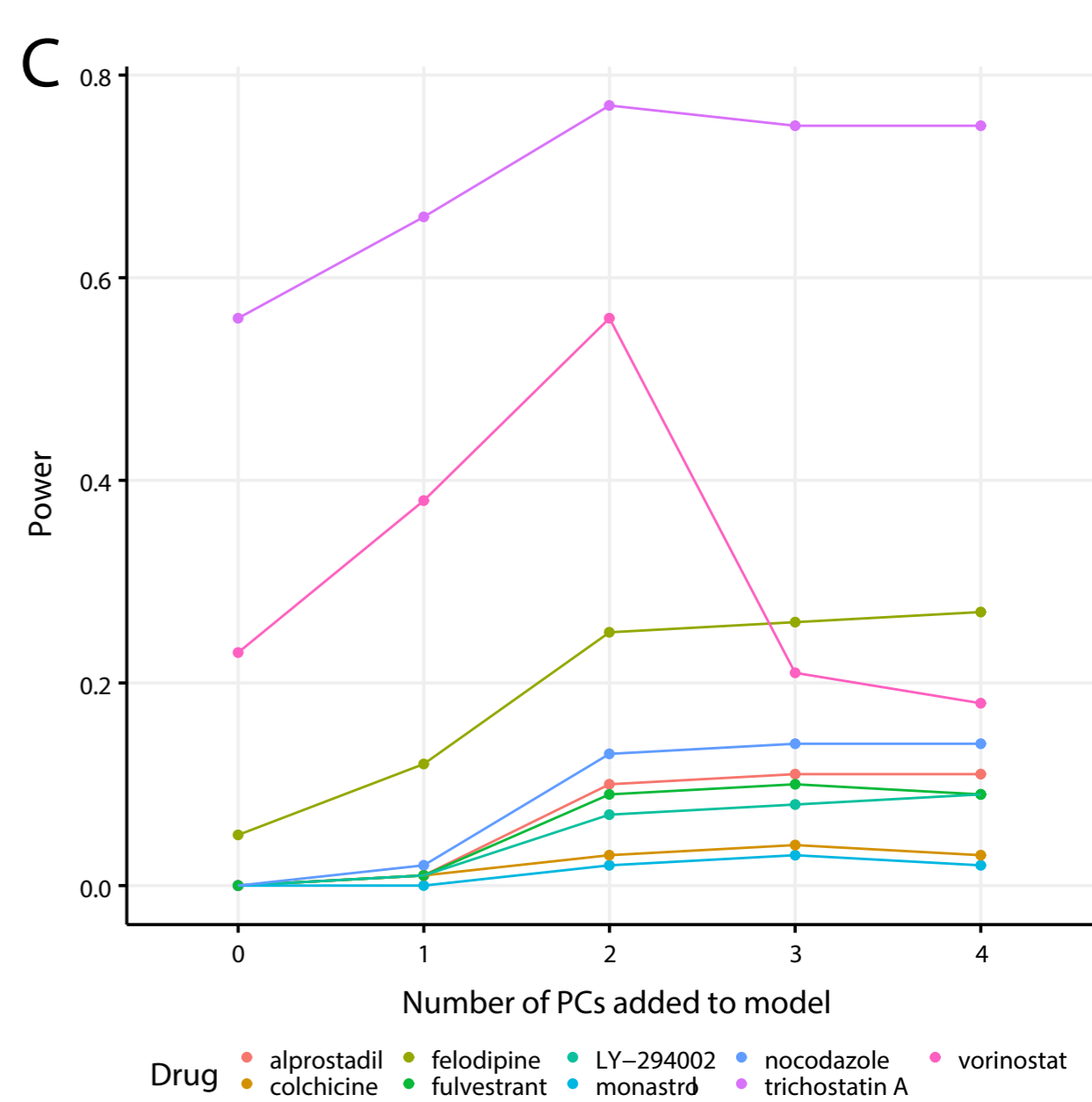

Supplement: Supplementary file 8 — Figure S8. Results of simulation study with medium batch effects and FDR < 10%. A, log10 transformed number of significant genes averaged over 10 simulations; B, Proportion of false positives among the significant genes averaged over 10 simulations; C. the power of the analysis averaged over 10 simulations. (PDF 95 kb) [file 12859_2019_3028_MOESM8_ESM.pdf]

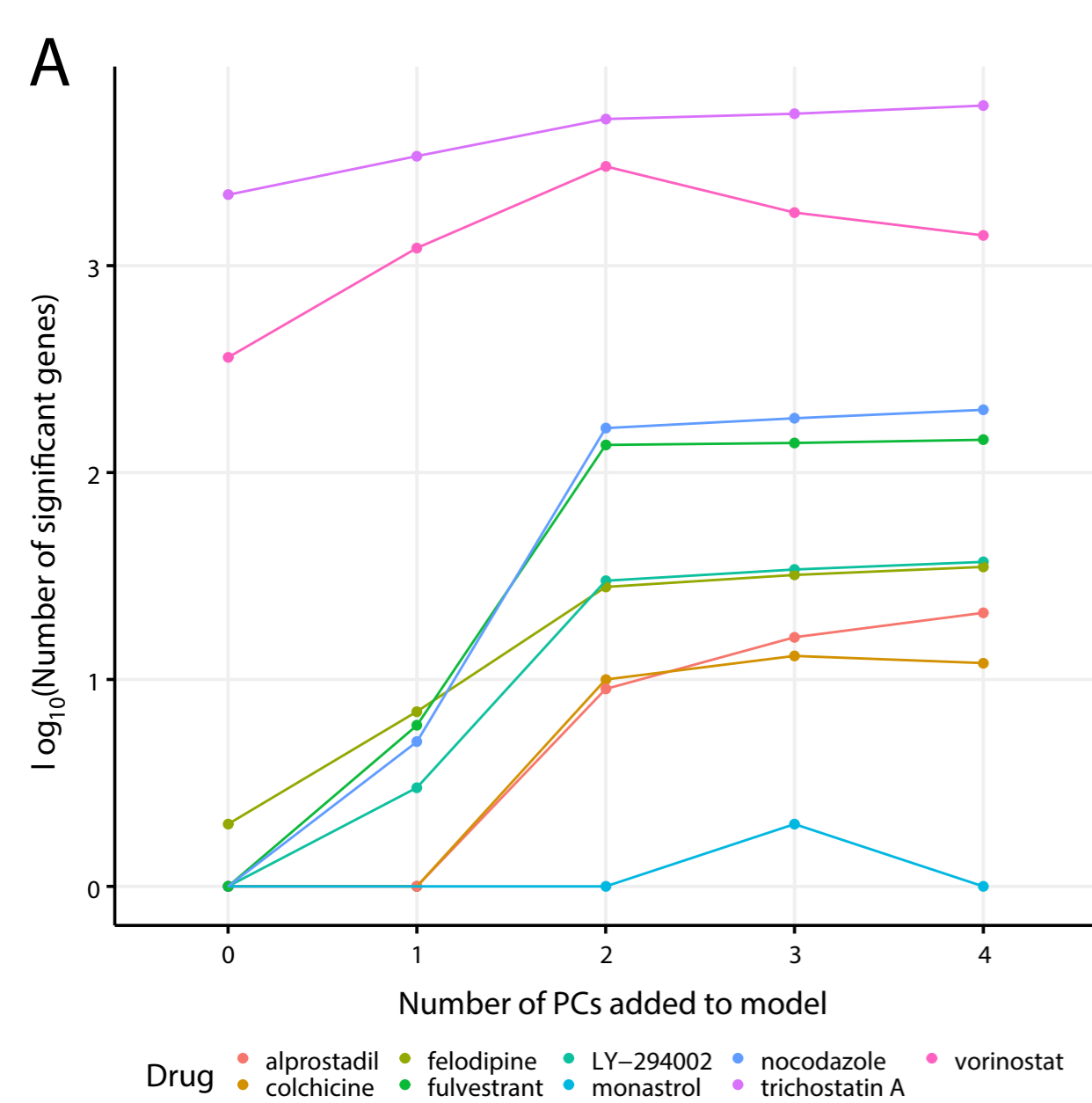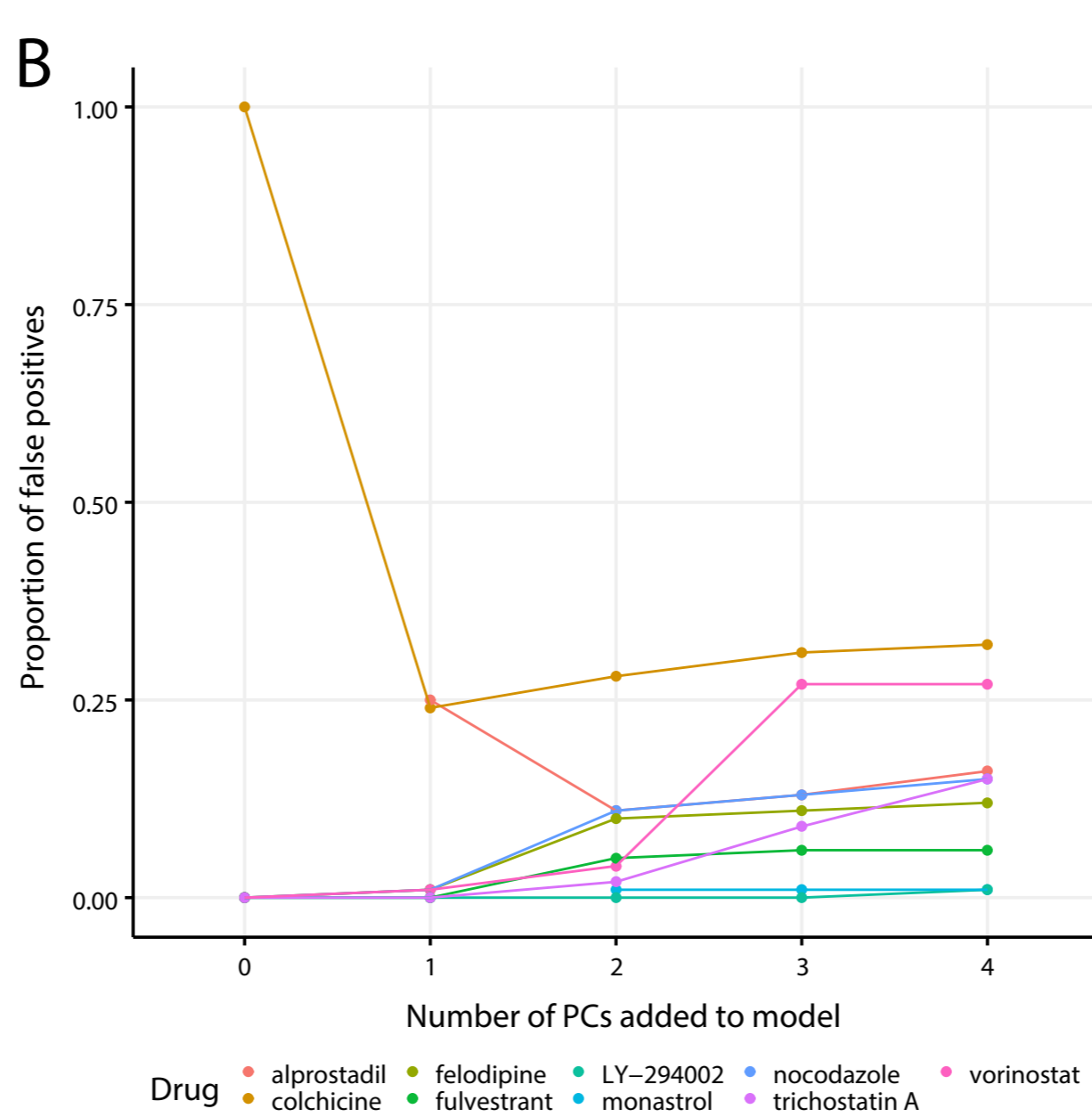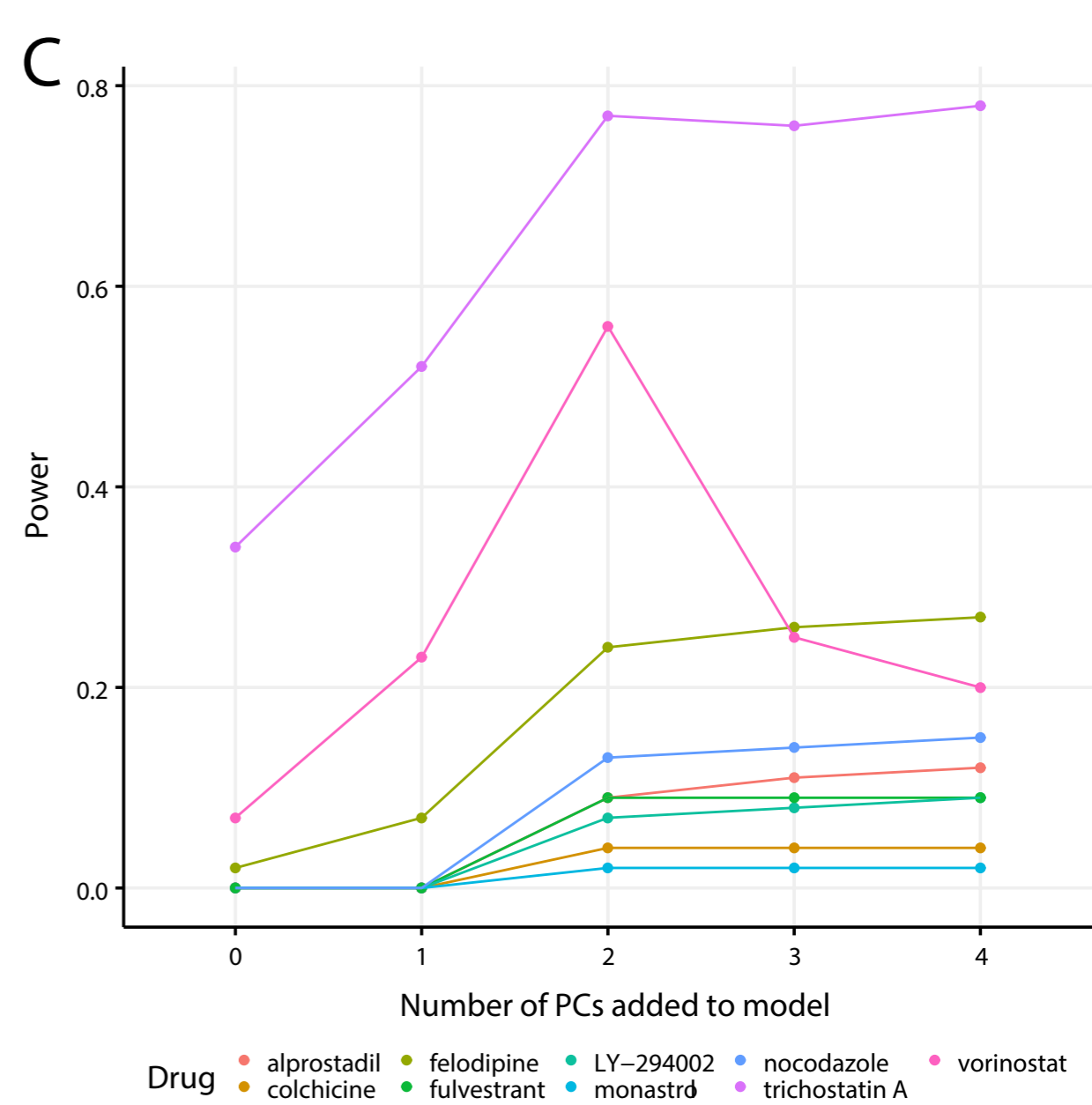

Supplement: Supplementary file 9 — Figure S9. Results of simulation study with large batch effects and FDR < 10%. A, log10 transformed number of significant genes averaged over 10 simulations; B, Proportion of false positives among the significant genes averaged over 10 simulations; C. the power of the analysis averaged over 10 simulations. (PDF 94 kb) [file 12859_2019_3028_MOESM9_ESM.pdf]

A

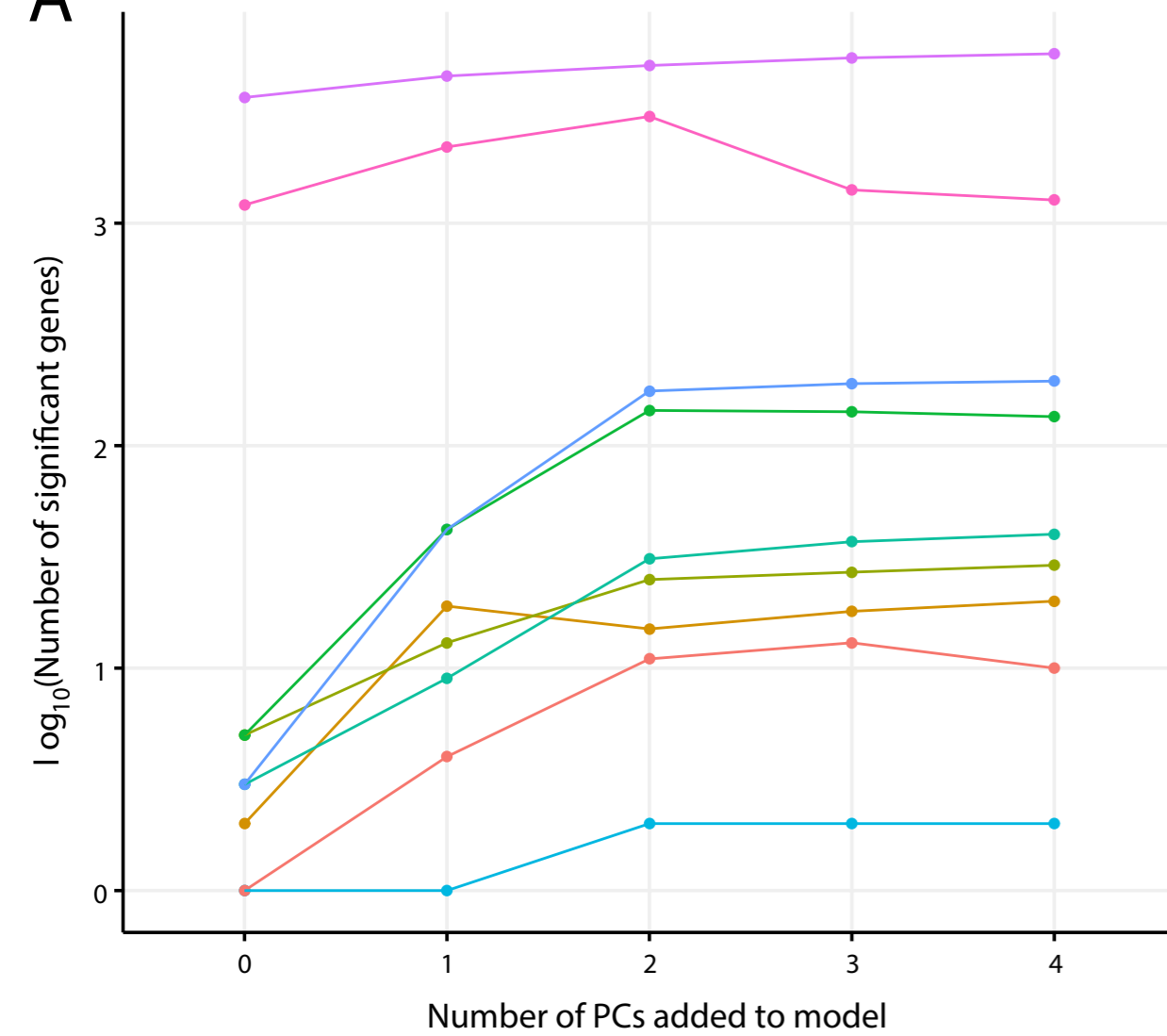

B

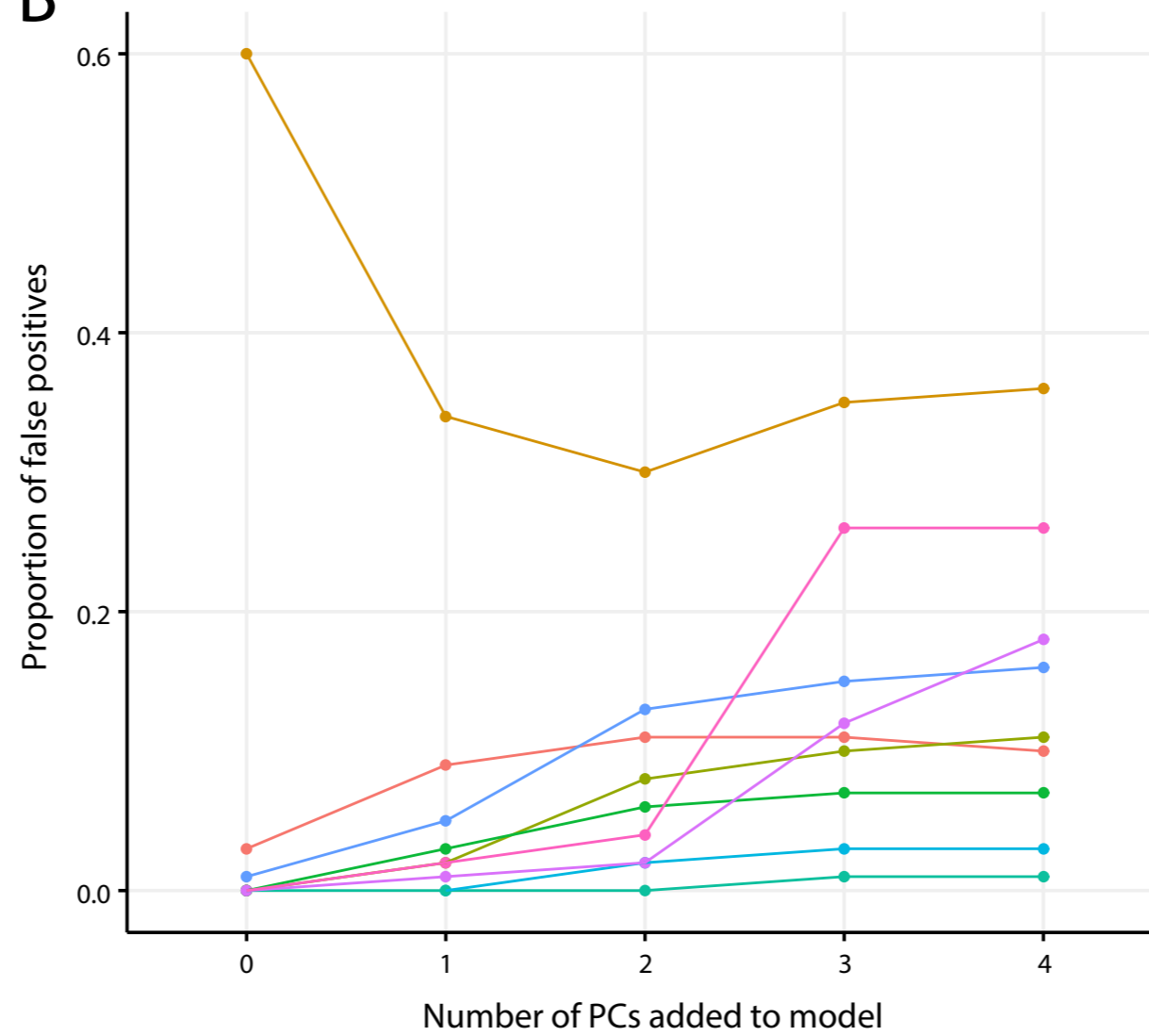

C

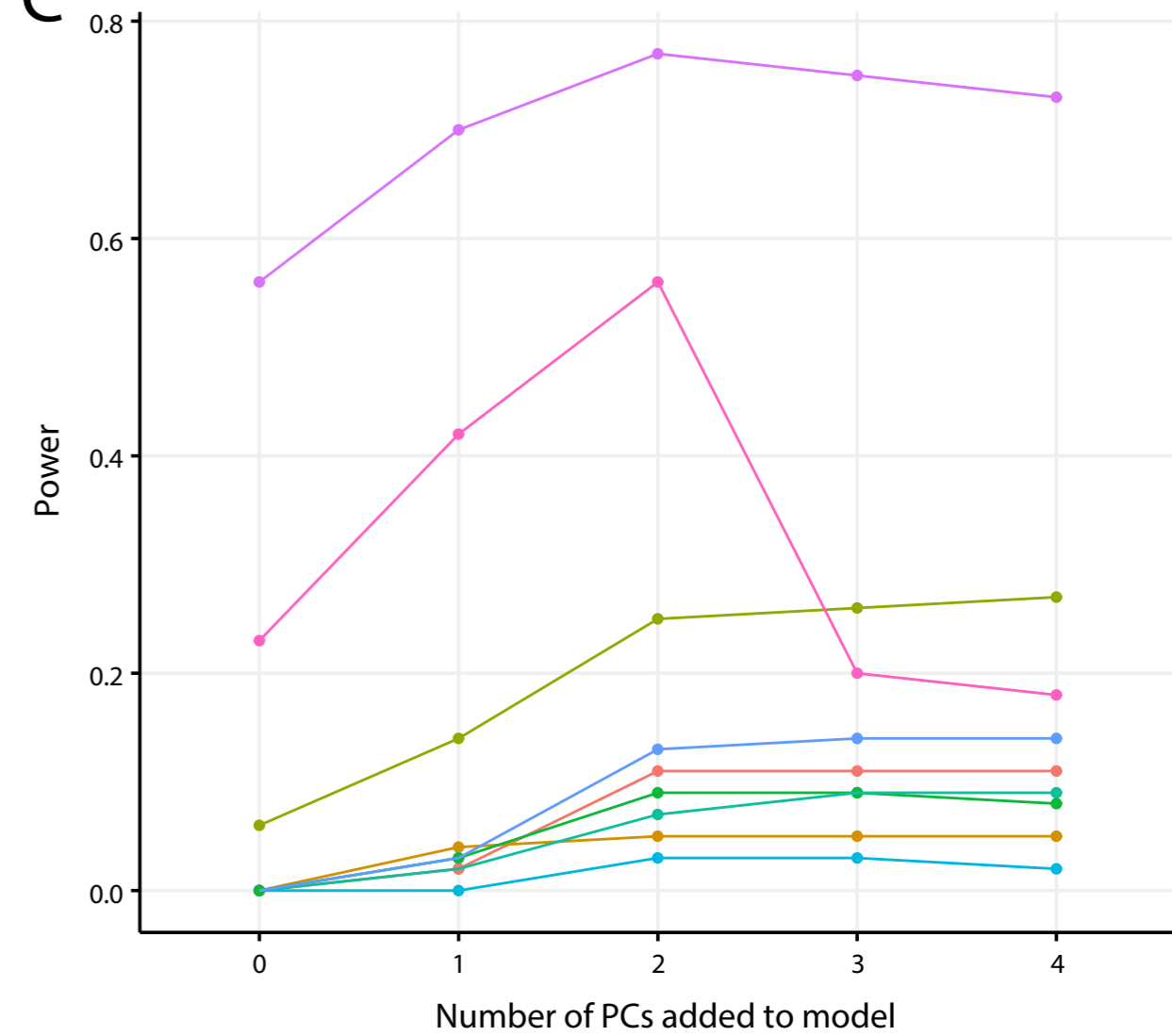

Supplement: Supplementary file 10 — Figure S10. Results of simulation study with medium batch effects, FDR < 10% and unequal allocation of cases and controls. Medium batch effect simulated with 0.3 differences between cases and controls. A, log10 transformed number of significant genes averaged over 10 simulations; B, Proportion of false positives among the significant genes averaged over 10 simulations; C. the power of the analysis averaged over 10 simulations. (PDF 94 kb) [file 12859_2019_3028_MOESM10_ESM.pdf]

**A**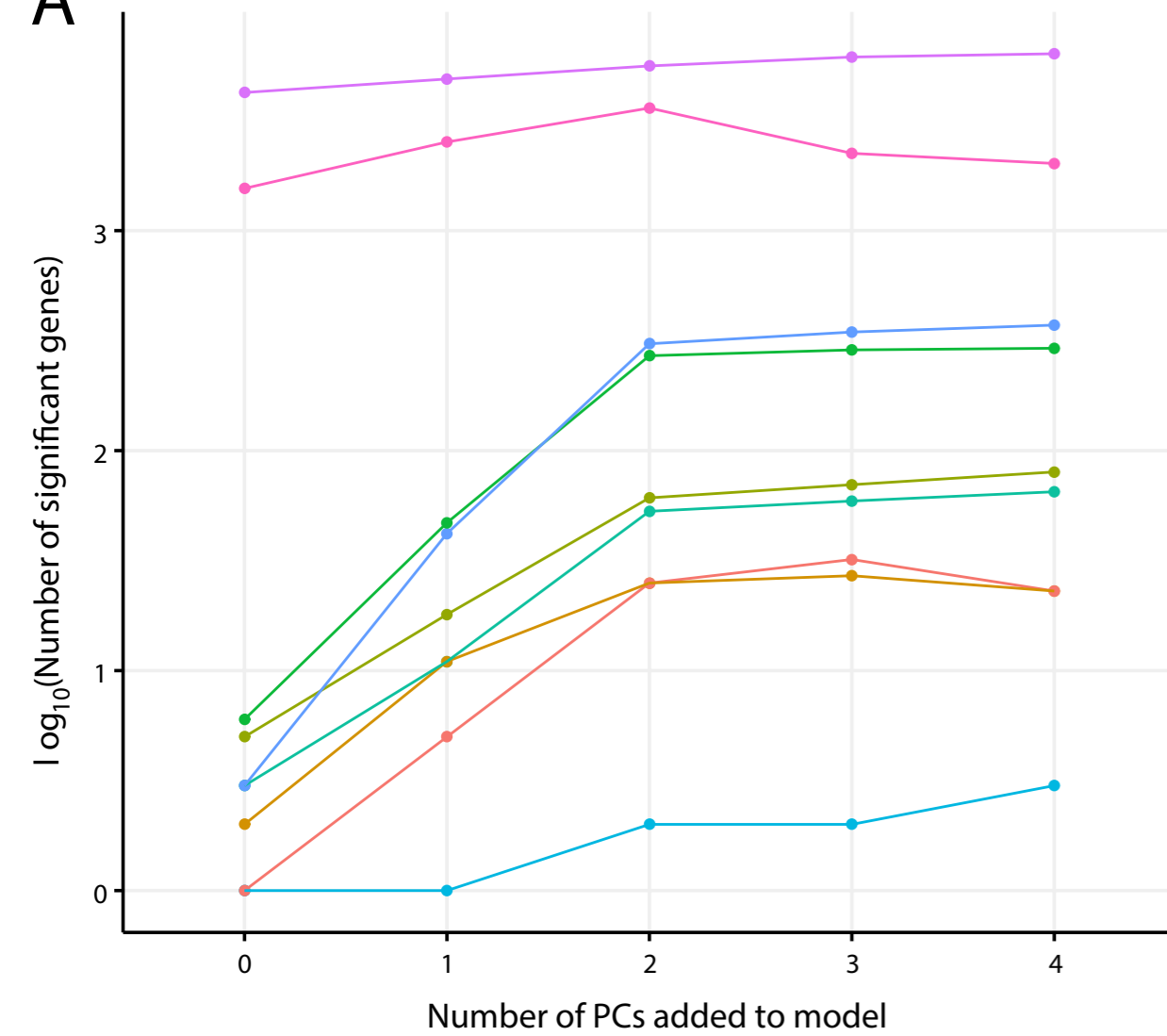**B**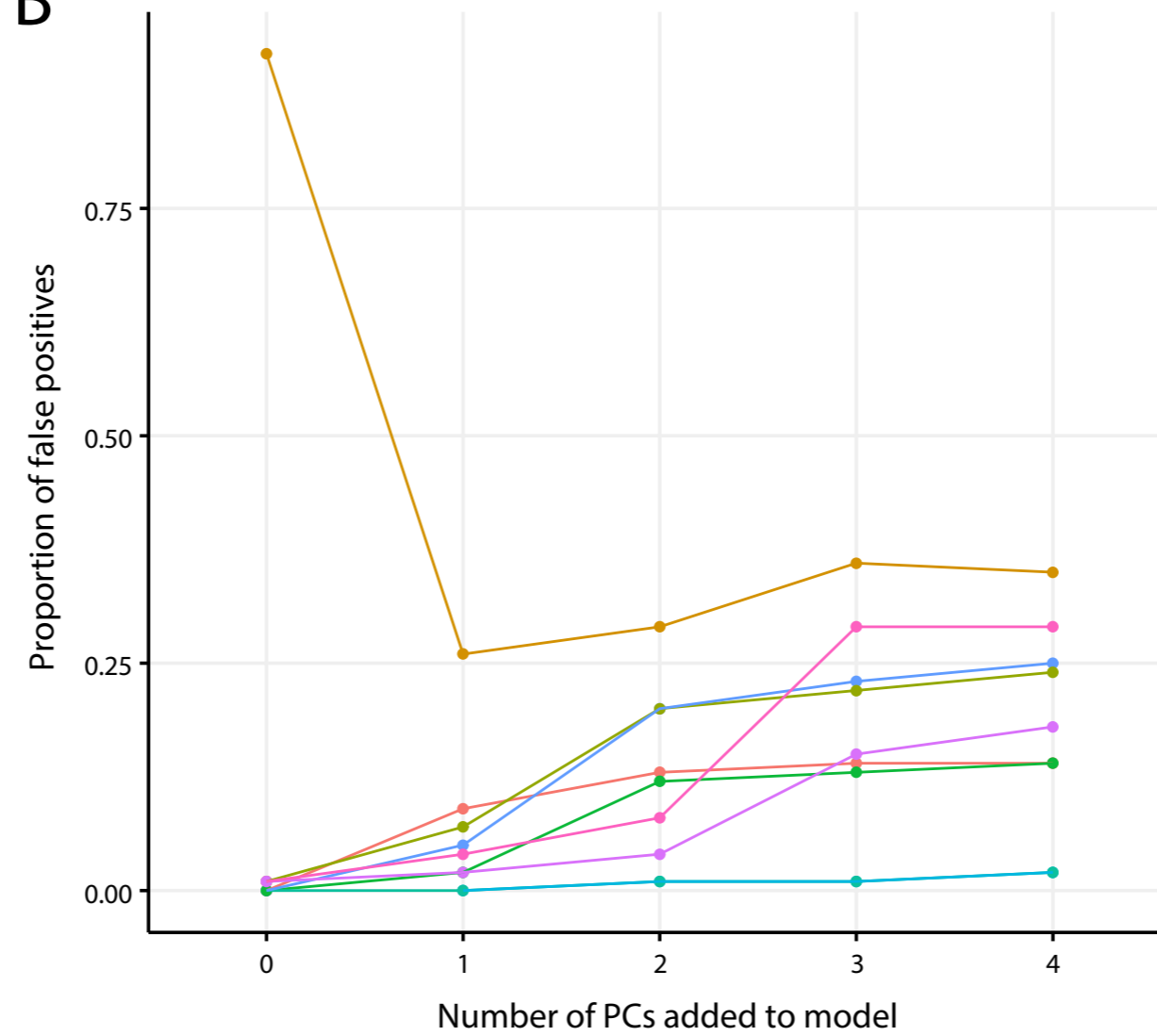**C**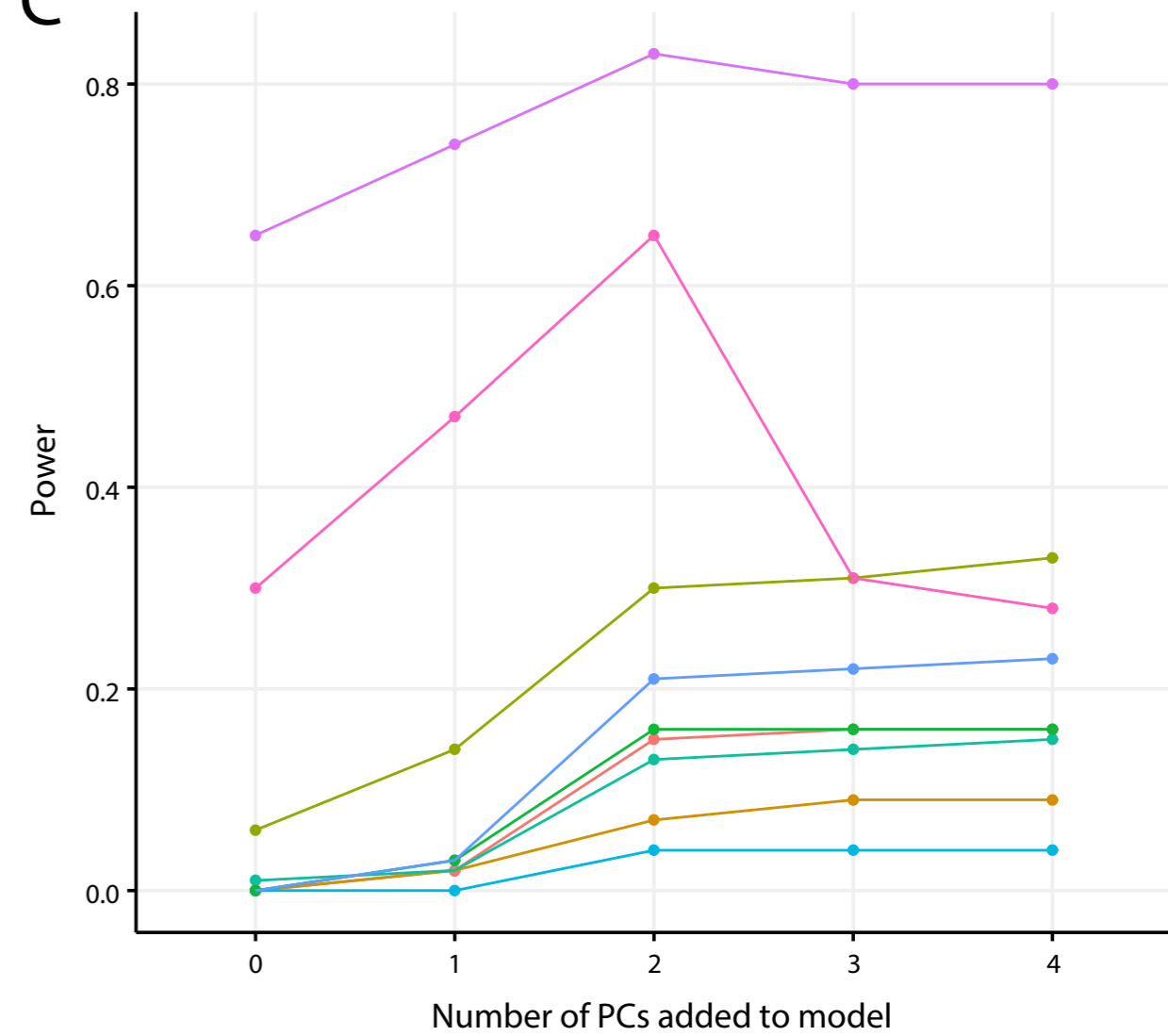

Supplement: Supplementary file 11 — Figure S11. Results of simulation study with medium batch effects and FDR < 20%. A, log10 transformed number of significant genes averaged over 10 simulations; B, Proportion of false positives among the significant genes averaged over 10 simulations; C. the power of the analysis averaged over 10 simulations. (PDF 94 kb) [file 12859_2019_3028_MOESM11_ESM.pdf]

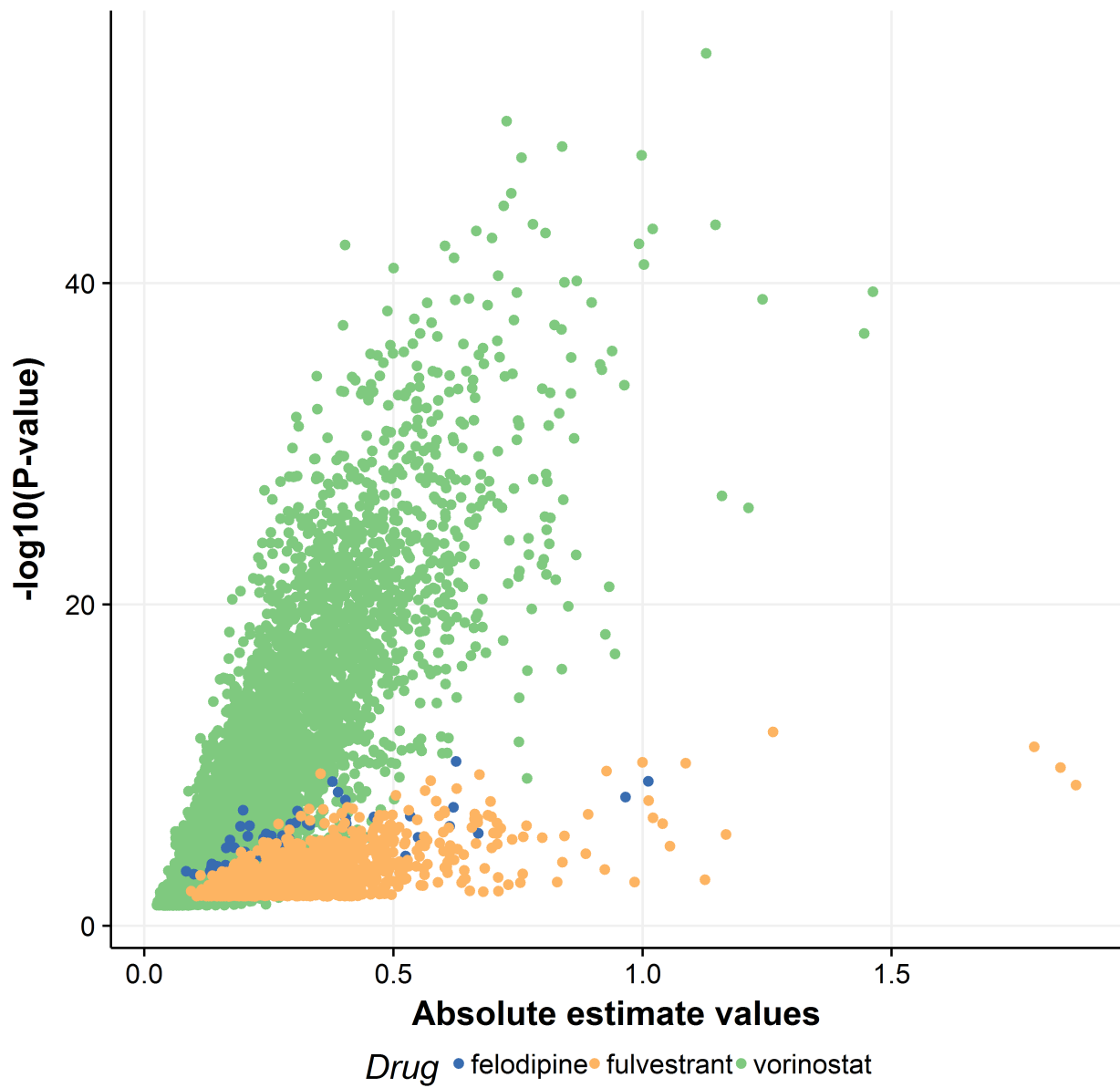

Supplement: Supplementary file 12 — Figure S12. Negative log 10 of P-values plotted against absolute estimates of extracted drug effects of felodipine, fulvestrant and vorinostat. (PDF 391 kb) [file 12859_2019_3028_MOESM12_ESM.pdf]
