# Supplementary material for: Influence of batch effect correction methods on drug induced differential gene expression profiles
Source: BMC Bioinformatics. 2019 Aug 22;20:437. doi: 10.1186/s12859-019-3028-6 (PMC6706913; doi:10.1186/s12859-019-3028-6)
Supplement: Supplementary file 14 — Figure S14. Histograms of P-values resulted from differential expression analyses on one set of data simulated from vorinostat with balanced batch design and median batch size (parameter settings see Table 4) at FDR ≤ 0.1. The differential expression analyses: A) limma + null model; B) limma + 1 PC; C) limma + 2 PCs; D) limma + 3 PCs; E) limma + 4 PCs. Abbreviations: PC, principal component; MPFP, mean proportion of false positive results. MPFP, mean proportion of false positives among the significant genes. (PDF 1242 kb) [file 12859_2019_3028_MOESM14_ESM.pdf]

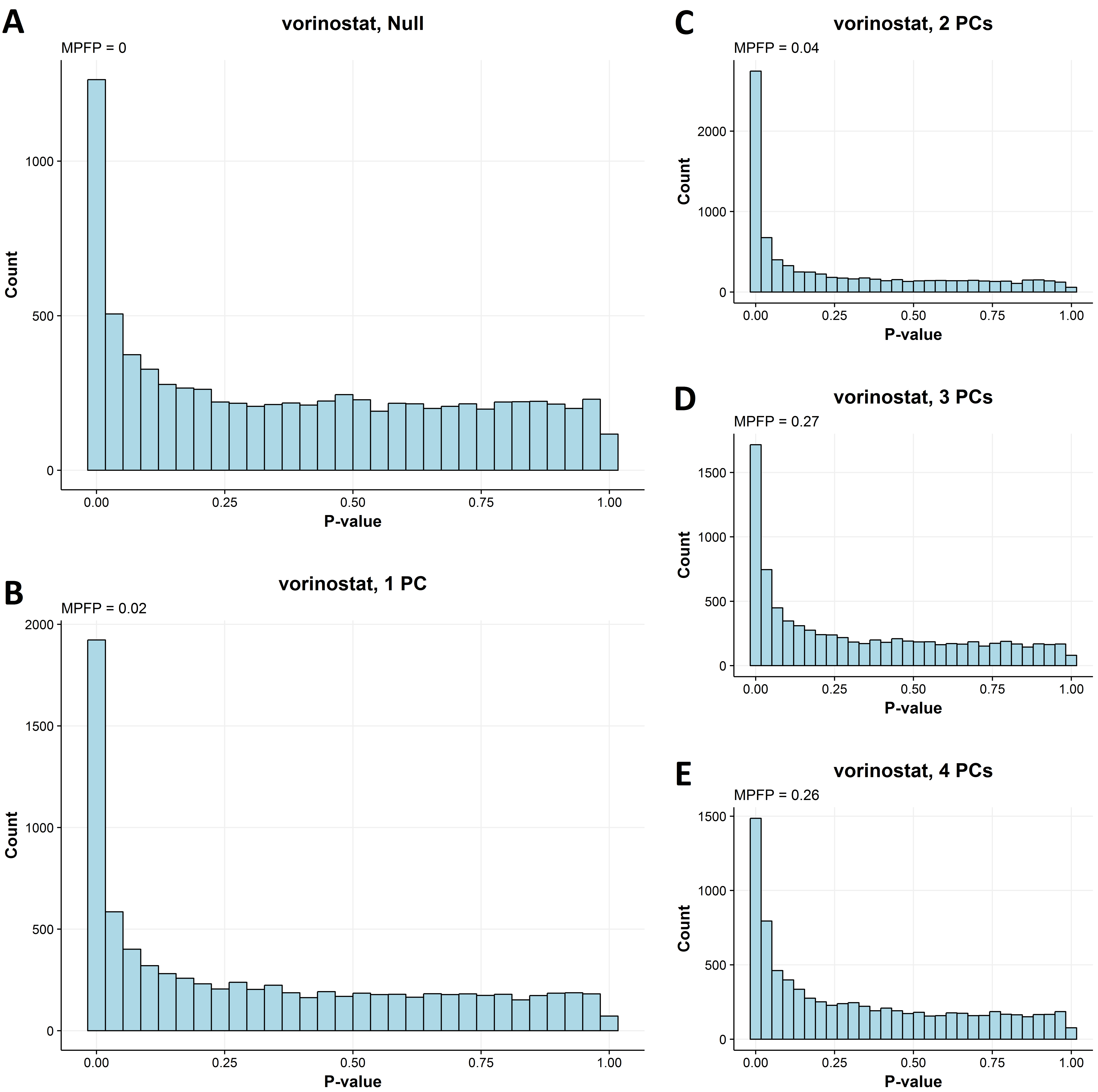

A
vorinostat, Null
MPFP = 0
Count
P-value
B
vorinostat, 1 PC
MPFP = 0.02
C
vorinostat, 2 PCs
MPFP = 0.04
D
vorinostat, 3 PCs
MPFP = 0.27
E
vorinostat, 4 PCs
MPFP = 0.26
